# Development of a novel Hsp90 inhibitor NCT-50 as a potential anticancer agent for the treatment of non-small cell lung cancer

**DOI:** 10.1038/s41598-018-32196-6

**Published:** 2018-09-17

**Authors:** Seung Yeob Hyun, Huong Thuy Le, Cong-Truong Nguyen, Young-Sik Yong, Hye-Jin Boo, Ho Jin Lee, Ji-Sun Lee, Hye-Young Min, Jihyae Ann, Jie Chen, Hyun-Ju Park, Jeewoo Lee, Ho-Young Lee

**Affiliations:** 1 Creative Research Initiative Center for concurrent control of emphysema and lung cancer, College of Pharmacy, Seoul National University, Seoul, 08826 Republic of Korea; 2 College of Pharmacy and Research Institute of Pharmaceutical Sciences, Seoul National University, Seoul, 08826 Republic of Korea; 3 Department of Molecular Medicine and Biopharmaceutical Sciences, Graduate School of Convergence Science and Technology, and College of Pharmacy, Seoul National University, Seoul, 08826 Republic of Korea; 4 School of Pharmacy, Sungkyunkwan University, Suwon, Gyeonggi-do 16419 South Korea

## Abstract

Despite the development of advanced therapeutic regimens such as molecular targeted therapy and immunotherapy, the 5-year survival of patients with lung cancer is still less than 20%, suggesting the need to develop additional treatment strategies. The molecular chaperone heat shock protein 90 (Hsp90) plays important roles in the maturation of oncogenic proteins and thus has been considered as an anticancer therapeutic target. Here we show the efficacy and biological mechanism of a Hsp90 inhibitor NCT-50, a novobiocin-deguelin analog hybridizing the pharmacophores of these known Hsp90 inhibitors. NCT-50 exhibited significant inhibitory effects on the viability and colony formation of non-small cell lung cancer (NSCLC) cells and those carrying resistance to chemotherapy. In contrast, NCT-50 showed minimal effects on the viability of normal cells. NCT-50 induced apoptosis in NSCLC cells, inhibited the expression and activity of several Hsp90 clients including hypoxia-inducible factor (HIF)-1α, and suppressed pro-angiogenic effects of NSCLC cells. Further biochemical and in silico studies revealed that NCT-50 downregulated Hsp90 function by interacting with the C-terminal ATP-binding pocket of Hsp90, leading to decrease in the interaction with Hsp90 client proteins. These results suggest the potential of NCT-50 as an anticancer Hsp90 inhibitor.

## Introduction

To maintain homeostasis during various extracellular and intracellular insults, cancer cells rely on heat shock protein 90 (Hsp90) to stabilize many proteins, constructing signaling networks responsible for cell survival, growth, and proliferation^1,2^. Indeed, Hsp90 client proteins are associated with the hallmarks of cancer^3,4^ and thus targeting Hsp90 has been considered an efficient anticancer therapeutic strategy^4^. Several Hsp90 inhibitors with various structural backbones have shown potent anticancer activities *in vitro* and *in vivo*^5^. However, none of these inhibitors have been approved for use in the clinic, and several clinical trials using Hsp90 inhibitors have been discontinued due to minimal effects and toxicity. Hence, it is necessary to develop safer and more effective Hsp90 inhibitors.

Lung cancer is the leading cause of cancer-related human deaths worldwide. Despite extensive efforts to develop efficient therapeutic interventions, the 5-year survival rate for lung cancer is less than 20%^6,7^. Based on several problems with chemotherapy, such as unselective toxicity, drug resistance, and recurrence, agents selectively targeting overactivated signaling pathways in cancer cells have been extensively developed and now several drugs are used as the first-line therapy for patients with lung cancer carrying relevant genetic alterations^8^. Although some therapies have initially shown remarkable anticancer effects^9,10^, recurrence due to drug resistance is an inevitable consequence of these targeted therapies in most cases. Thus, there is an urgent unmet need to develop more efficacious anticancer agents that can be utilized as a monotherapy or an adjuvant therapy with other anticancer drugs.

Natural products are considered an important source for the development of anticancer drugs^11^. We have demonstrated the anticancer and cancer chemopreventive effects of a naturally occurring rotenoid deguelin^12–17^. Further studies revealed Hsp90 as the molecular target for deguelin’s antitumor effect^18^. However, the potential toxic effects of deguelin, such as a Parkinson’s disease-like syndrome^19^, hamper the further clinical utility of deguelin. Novobiocin, another natural product, is derived from Streptomyces and is a coumarin antibiotics with Hsp90 inhibiting activity^2,20,21^. However, it’s half maximal inhibitory concentration (IC_50_) has been reported to be up to 700 μM^22^, making it difficult to utilize as a clinical therapeutic.

**Figure 1:**
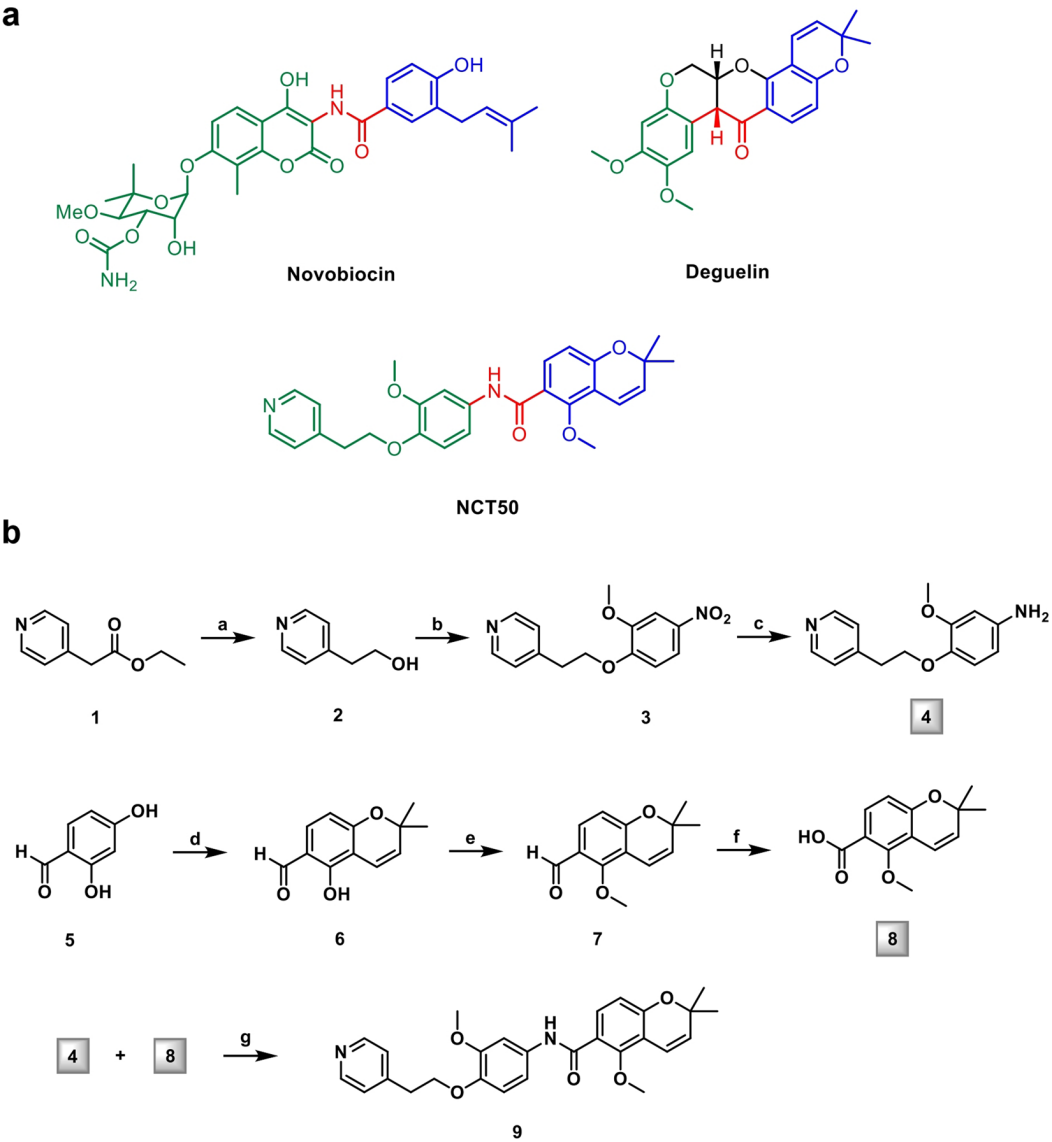
Synthesis of NCT-50. (**a**) The chemical structures of novobiocin, deguelin, and NCT-50. (**b**) Synthetic scheme of NCT-50.

To circumvent the limitation of deguelin, we have developed several deguelin analogs that harbor less or no potential toxicity to various normal cells^16,23–27^. We and others previously demonstrate the involvement of the dimethoxy benzopyran moiety of deguelin in the interaction with Hsp90^18^ and the crucial association of the acylamino moiety with the antiproliferative activity of novobiocin^28^. Importantly, the structural features of deguelin and novobiocin, which are highlighted in three different colors, indicate that they share principal pharmacophores (Fig. 1a). Thus, in addition to our previous efforts on the development of ring-truncated deguelin analogs^23–27^, here we synthesized a novel novobiocin-deguelin analog, designated 5-methoxy-N-(3-methoxy-4-(2-(pyridin-4-yl)ethoxy)phenyl)-2,2-dimethyl-2H-chromene-6-carboxamide (NCT-50), by hybridizing pharmacophores of these compounds and evaluated the effects of NCT-50 on the viability of non-small cell lung cancer (NSCLC) cells, which account for 80–85% of lung cancer^29^. NCT-50 displayed significant inhibitory effects on the viability and colony formation of NSCLC cells with minimal effects on the viability of normal cells. NCT-50 also induced apoptosis in NSCLC cells and inhibited pro-angiogenic activities of NSCLC cells. Further mechanistic investigation revealed that NCT-50 disrupted Hsp90 function by interacting with the ATP-binding pocket in the C-terminal domain of Hsp90 and concomitantly suppressed the expression and activity of multiple Hsp90 clients, including hypoxia-inducible factor (HIF)-1α. These results demonstrate the potential use of NCT-50 as an Hsp90-targeting anticancer agent.

## Results

### Synthesis of NCT-50

NCT-50 is a derivative designed as a simplified hybrid of novobiocin and deguelin to form the three principal pharmacophores. Structurally, NCT-50 has 2,2-dimethyl-2H-chromene and 1-methoxy-2-((pyridin-4-yl)ethoxy)phenyl rings linked by an amide bond (Fig. 1a). Two key intermediates, the aniline in the left part (4) and the carboxylic acid of the chromene moiety (8), were synthesized and then coupled to each other (Fig. 1b).

### NCT-50 significantly inhibits the viability and colony-forming ability of NSCLC cells with minimal effect on the viability of normal cells

**Figure 2:**
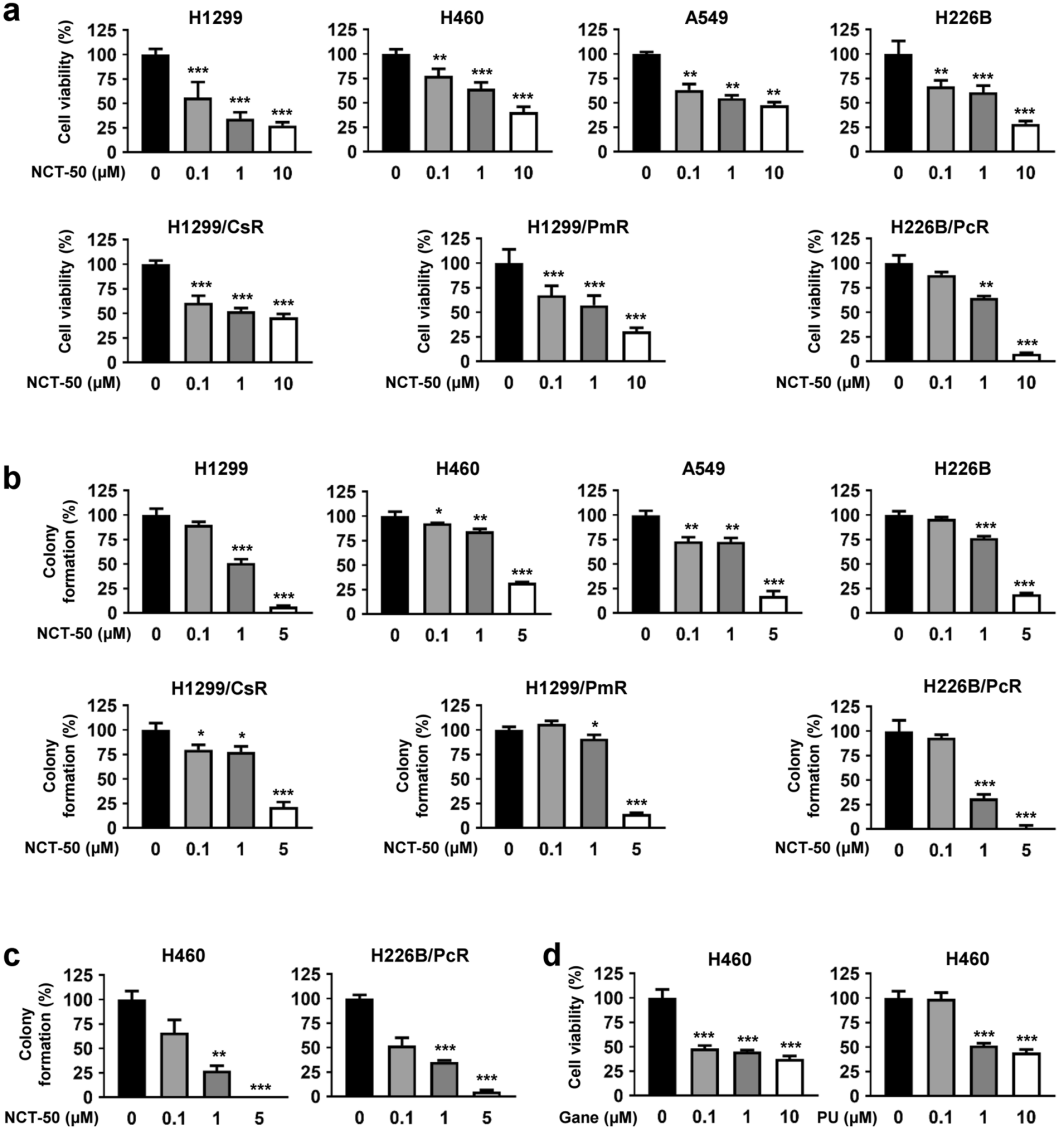
Effects of NCT-50 on the viability and colony formation of NSCLC cells. (**a**) NSCLC (H1299, H460, A549 and H226B) cells and those carrying resistance to cisplatin (H1299/CsR), pemetrexed (H1299/PmR), and paclitaxel (H226B/PcR) were treated with increasing concentrations of NCT-50 (**a**) for 3 days. Cell viability was determined by the MTT assay. (**b**,**c**) The anchorage-dependent (**b**) and -independent (**c**) colony formation of NSCLC cells treated with increasing concentrations of NCT-50 was determined as described in Methods. (**d**) H460 cells were treated with increasing concentrations of ganetespib (Gane) or PU-H71 (PU) for 3 days. Cell viability was determined by the MTT assay. The bars represent the mean ± SD. ^*^P < 0.05, ^**^P < 0.01, and ^***^P < 0.001 by Student’s t-test compared with vehicle-treated control group.

We then examined the effect of NCT-50 on the viability and colony formation of human NSCLC cells, either naïve or resistant to anticancer drugs^23^, considering that resistance to chemotherapy is a large obstacle for efficient anticancer therapeutics. NCT-50 significantly inhibited the viability and anchorage-dependent colony formation of NSCLC cells in a concentration-dependent manner (Fig. 2a,b). The IC_50_ values of NCT-50 on the inhibition of NSCLC cell viability were about 2 μM on average (Table 1), which was comparable or superior to previously developed deguelin analogs^23,24,26^, novobiocin, and its analogs^22,28,30^. The inhibitory effect of NCT-50 on the viability and anchorage-dependent colony formation of drug-resistant sublines (H1299/CsR, H1299/PmR, and H226B/PcR) was comparable with that on the corresponding naïve cells (H1299 and H226B), suggesting the effectiveness of NCT-50 for both chemo-naïve and chemo-resistant NSCLC cells (Fig. 2a,b). Consistent with these results, the colony formation of NSCLC cells (either chemo-naïve or chemoresistant), under anchorage-independent culture conditions, was also markedly suppressed by treatment with NCT-50, especially at the concentration of 5 μM (Fig. 2c), indicating the effectiveness of NCT-50 in suppressing the tumorigenicity of NSCLC cells, regardless of their drug resistance status. NCT-50 displayed weak but comparable cytotoxic effects in NSCLC cells compared with known Hsp90 inhibitors that have been evaluated in clinical trials such as ganetespib and PU-H71^31,32^ (Fig. 2d). These results suggest that NCT-50 suppresses the survival and the colony-forming ability of both chemo-naïve and chemo-resistant NSCLC cells.

**Table 1:** The IC_50_ values showing the inhibitory effects of NCT-50 on the cell viability.

Type	Name	IC_50_ (μM)
NSCLC cells	H1299	0.23
	H460	3.44
	A549	2.22
	H226B	1.63
	H1299/CsR	1.47
	H1299/PmR	1.42
	H226B/PcR	1.54
Retinal pigment epithelial cells	RPE	>10
Human umbilical vein endothelial cells	HUVEC	>10
Human lung epithelial cells	HBE	>10
	BEAS-2B	>10
Murine hippocampal neuronal cells	HT-22	>10

The IC_50_ values of NCT-50 for the suppression of the viability of indicated cells were calculated by non-linear regression analysis.

### Induction of apoptosis is associated with NCT-50-medated suppression of NSCLC cell viability

**Figure 3:**
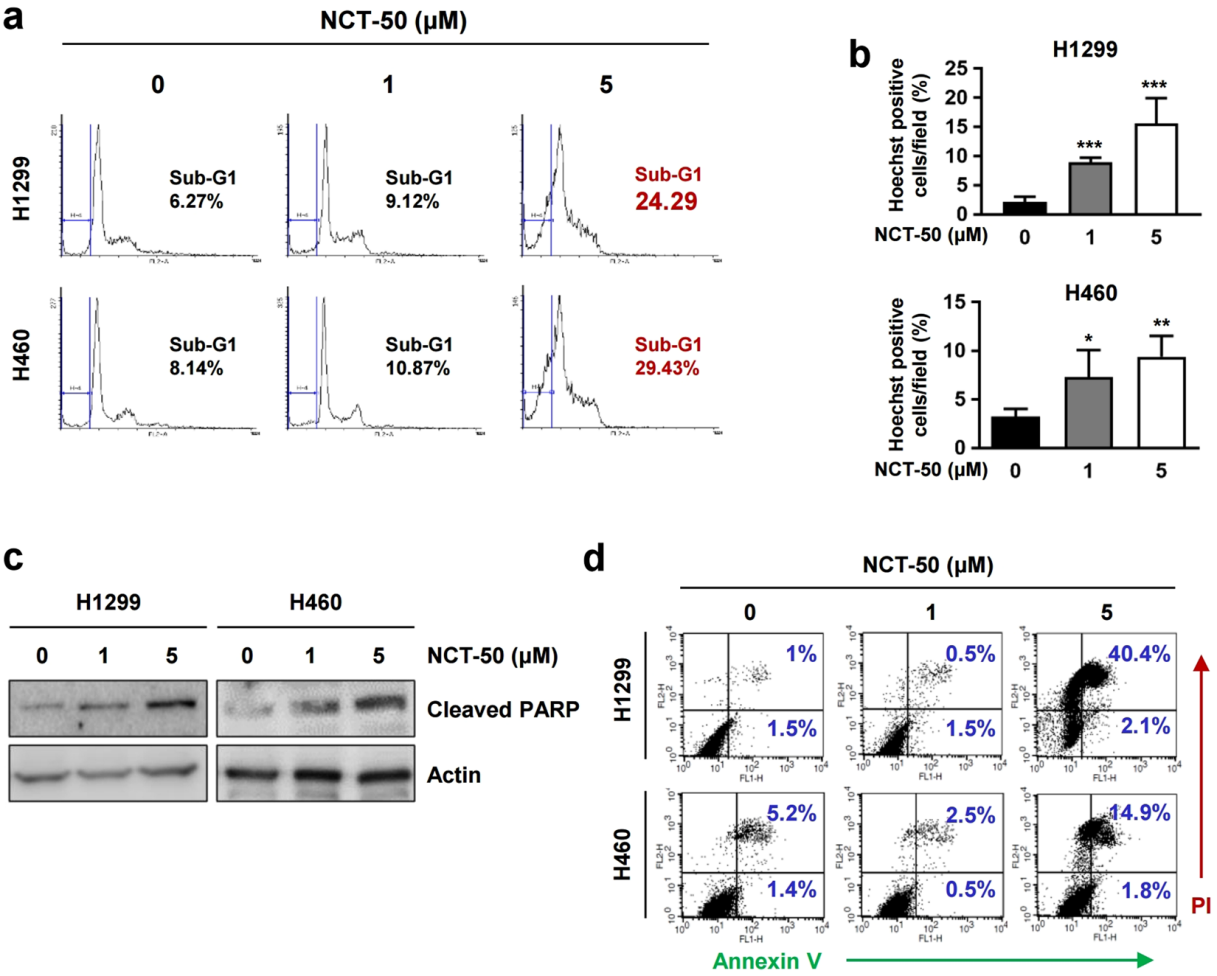
Association of apoptosis with NCT-50-induced cell death. (**a–d**) H1299 and H460 cells were treated with NCT-50 for 2 days. (**a**) The distribution of cells in each phase of the cell cycle was analyzed by flow cytometry. (**b**) Condensed, fragmented or degraded nuclei was analyzed by Hoechst 33258 staining and counted. (**c**) The level of cleaved PARP expression was analyzed by Western blot analysis. (**d**) The annexin V-positive cell population was determined by flow cytometry as described in Methods. The bars represent the mean ± SD. ^*^P < 0.05, ^**^P < 0.01, and ^***^P < 0.001 by Student’s t-test compared with vehicle-treated control group.

According to the efficient inhibitory effects of NCT-50 on the viability and colony-forming ability of NSCLC cells, we investigated the underlying mechanism. First, using flow cytometry, we examined the effect of NCT-50 on cell cycle progression. As shown in Fig. 3a, approximately 30% of the cell population was accumulated in the sub-G1 phase in H1299 and H460 cells after treatment with 5 μM NCT-50. Hoechst 33258 staining also showed increased chromatin condensation, a feature of apoptotic cells^33^, in NCT-50-treated cells (Fig. 3b). Western blot analysis also revealed obviously increased levels of poly-(ADP-ribose) polymerase (PARP) cleavage in the two cell lines after the drug treatment (Fig. 3c). Consistently, the Annexin V-positive population was markedly increased in the drug-treated cells (Fig. 3d). These results indicated apoptotic activities of NCT-50 in NSCLC cells, suggesting that the reduced NSCLC cell viability after treatment with NCT-50 is due, in part, to increased apoptosis.

### NCT-50 displays improved safety profiles compared with known Hsp90 inhibitors and deguelin

**Figure 4:**
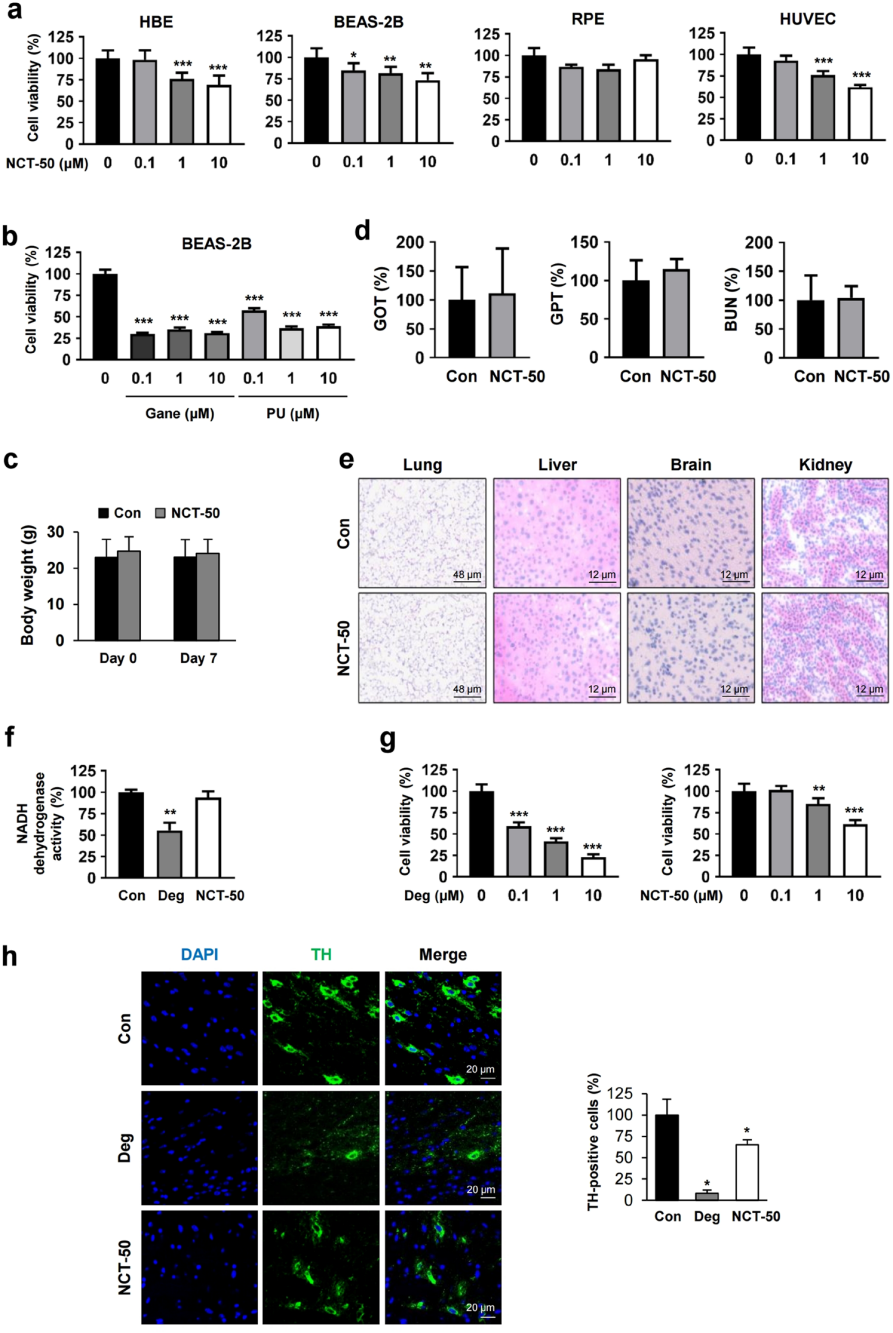
Improved safety of NCT-50 compared with known Hsp90 inhibitors and deguelin. (**a**) Various normal cells were treated with vehicle (DMSO) or NCT-50 (0.1, 1, and 10 μM) for 3 days. Cell viability was determined by the MTT assay. (**b**) BEAS-2B cells were treated with increasing concentrations of Hsp90 inhibitors [ganetespib (Gane) or PU-H71 (PU)] for 2 days. Cell viability was determined by the MTT assay. (**c**) Body weight changes between vehicle- (control) and NCT-50-treated mice. (**d**) The level of GOT, GPT, and BUN in the serum was determined as described in Methods and expressed as a percentage of vehicle-treated control group. (**e**) The histopathological changes in liver, lung, brain, and kidney from mice treated with vehicle or NCT-50 were evaluated by H&E-stained section of the tissues. The representative images were shown. (**f**) Spectrophotometric analysis of NADH dehydrogenase activity using mitochondria-enriched fractions was performed as described in Methods. (**g**) HT-22 cells were treated with various concentrations of deguelin or NCT-50 for 2 days. Cell viability was determined by the MTT assay. (**h**) Representative images showing tyrosine hydroxylase immunoreactivity in the midbrain from vehicle, deguelin, or NCT-50-treated mice. *Right*. Quantitative analysis of tyrosine hydroxylase immunoreactivity in each group, expressed as a percentage of vehicle-treated control group. The bars represent the mean ± SD. ^*^P < 0.05, ^**^P < 0.01, and ^***^P < 0.001 by Student’s t-test compared with vehicle-treated control group.

Considering the potential toxic effect of Hsp90 inhibitors^34^ and deguelin^19^, we next examined whether NCT-50 has improved safety profiles compared to these known Hsp90 inhibitors. First, we assessed cytotoxicity of NCT-50 at the cellular levels by testing the effects of NCT-50 on the viability of several normal cells, including two human normal lung epithelial cells (HBE and BEAS-2B), human retinal pigment epithelial cells (RPE), and human vascular endothelial cells (HUVECs). Compared to various cancer cell lines treated with NCT-50 (Fig. 2), these normal cell lines showed minimal changes in their viability after the treatment with NCT-50 (10 μM) (Fig. 4a). The IC_50_ values of NCT-50 on the viability of these normal cells was over 10 μM (Table 1). Moreover, in contrast to minimal cytotoxic effect of NCT-50, known Hsp90 inhibitors such as ganetespib and PU-H71 displayed remarkable cytotoxic effects in BEAS-2B cells, suggesting reduced toxicity of NCT-50 compared with these Hsp90 inhibitors (Fig. 4b). We further performed several *in vivo* experiments to evaluate toxicity profiles of NCT-50. Mice in a FVB background were orally administered with 4 mg/kg NCT-50 twice a day for 7 consecutive days. Compared with vehicle-treated mice, NCT-50-treated mice displayed no significant changes in body weight (Fig. 4c). The serum levels GOT (glutamate oxaloacetate transaminase), GPT (glutamate pyruvate transaminase), and blood urea nitrogen (BUN), indicators of liver and renal function^35,36^, were not significantly different between vehicle- and NCT-50-treated mice (Fig. 4d). Moreover, histological analyses of H&E-stained tissue samples obtained from several organs (lung, liver, brain, and kidney) of NCT-50-treated mice revealed no remarkable histopathological changes (Fig. 4e). These results collectively indicate minimal toxicities of NCT-50.

According to the concern that deguelin may induce Parkinson’s-like syndrome^19^, which was manifested by decreased tyrosine hydroxylase immunoreactivity in the rat brain^19^, we evaluated the potential neurotoxicity of NCT-50. Because inhibition of NADH dehydrogenase activity was responsible for the potential neurotoxicity of deguelin^19^, we first determined the effects of NCT-50 and deguelin on NADH dehydrogenase activity using a mitochondria enriched fraction. In contrast to the significant inhibitory effects of deguelin, NCT-50 showed no detectable impact on the mitochondrial NADH dehydrogenase activity (Fig. 4f). We further compared the effect of NCT-50 and deguelin on the viability of mouse hippocampal neuronal cell line (HT-22). Compared to deguelin, NCT-50 induced minimal changes in HT-22 cells viability (Fig. 4g).

To verify these *in vitro* results, we determined *in vivo* neurotoxicity of NCT-50. To this end, mice were orally administered with NCT-50 or deguelin (4 mg/kg) twice a day for 7 consecutive days. We compared the effects of NCT-50 and deguelin on the immunoreactivity of tyrosine hydroxylase (TH), an enzyme in the late-limiting step of dopamine synthesis that has been used as a marker of dopaminergic neuron^37,38^, in the mouse midbrain. Consistent with the previous findings in the rat brain^19,25^, the TH immunoreactivity was significantly decreased by deguelin treatment in the mouse midbrain (Fig. 4h). In contrast, NCT-50 treatment minimally altered the level of the TH immunoreactivity. Taken together, these results indicate the markedly improved safety profile of NCT-50 compared with deguelin.

### NCT-50 inhibits expression of client proteins of Hsp90 and shows anti-angiogenic activities

**Figure 5:**
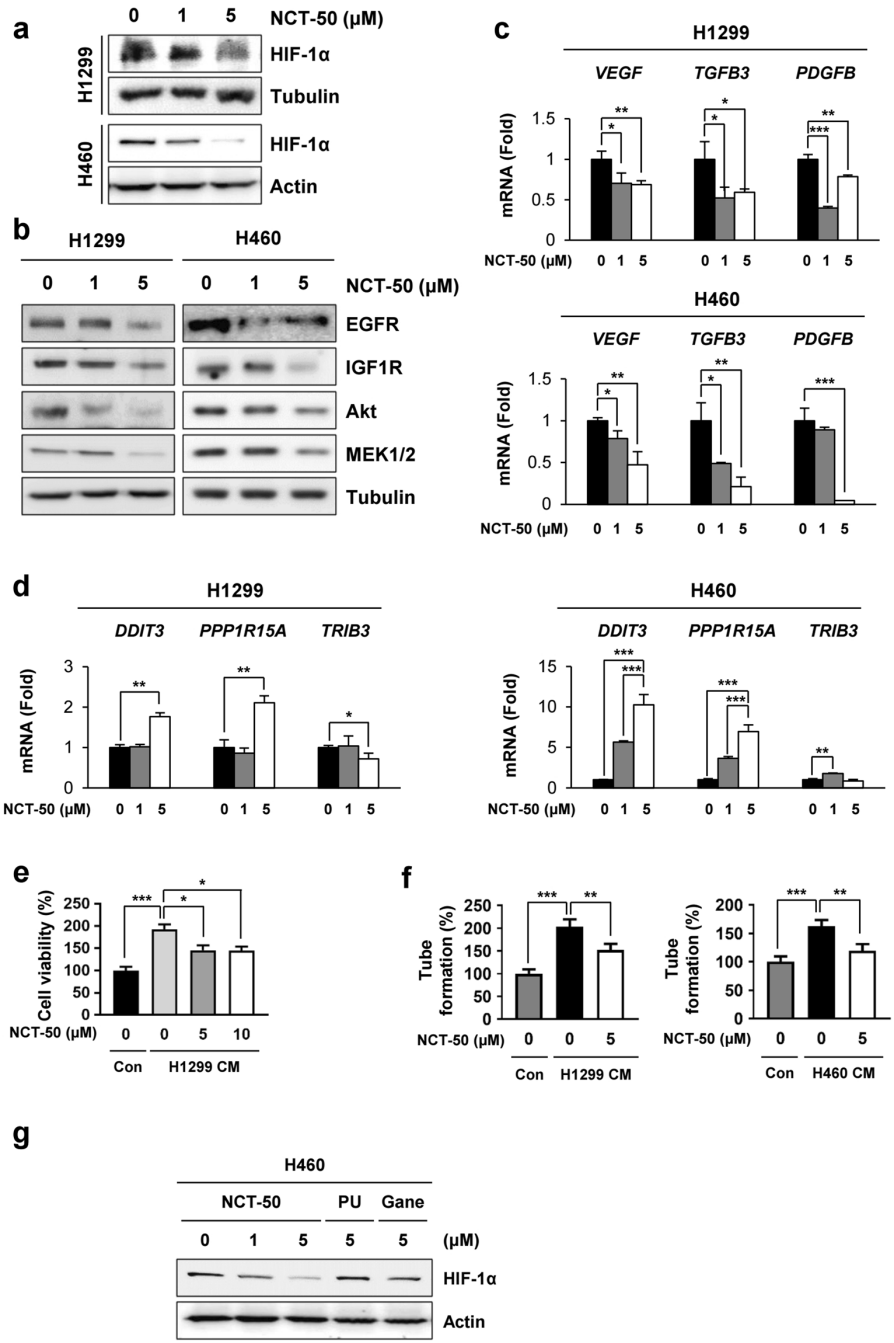
Downregulation of Hsp90 function by treatment with NCT-50. (**a**,**b**) H1299 and H460 cells were treated with NCT-50 for 24 h under hypoxic (**a**) or normoxic (**b**) conditions. The level of Hsp90 client proteins such as HIF-1α (**a**), EGFR, IGF1R, Akt, and MEK1/2 (**b**) was determined by Western blot analysis. (**c**,**d**) H1299 and H460 cells were treated with NCT-50 for 24 h. The mRNA expression of HIF-1α target genes (*VEGF*, *TGFB3*, and *PDGFB*) (**c**) or non-HIF-1α target genes (*DDIT3*, *PPP1R15A*, and *TRIB3*) (**d**) were determined by real-time PCR analysis. (**e**,**f**) HUVECs were treated with CM obtained from NSCLC cells treated with vehicle or NCT-50 (5 or 10 μM). The proliferation (**e**) and tube formation (**f**) of HUVECs were determined as described in Methods. (**g**) H460 cells were treated with NCT-50, ganetespib (Gane) or PU-H71 (PU) for 24 h under hypoxic conditions. The level of HIF-1α expression was determined by Western blot analysis. The bars represent the mean ± SD. ^*^P < 0.05, ^**^P < 0.01, and ^***^P < 0.001 by Student’s t-test compared with vehicle-treated control group.

Based on the previous studies demonstrating the inhibitory effect of novobiocin^20^ and deguelin^18^, we assessed whether NCT-50 could suppress expression of Hsp90 client proteins. Treatment with NCT-50 in hypoxic conditions decreased HIF-1α expression in a dose-dependent manner (Fig. 5a). The NCT treatment also inhibited the expression of several Hsp90 client proteins, including epidermal growth factor receptor (EGFR), insulin-like growth factor receptor-1 (IGF-1R), Akt, and MEK1/2^4,39^ in normoxic conditions. Moreover, NCT-50 markedly suppressed the expression of HIF-1α target genes (*VEGF*, *TGFB*3, and *PDGFB*^40^) (Fig. 5b) but not non-HIF target genes [*DDIT3* (encoding GADD153/CHOP)^41^, *PPPIR15A* (a GADD153-target gene^42^), and *TRIB3*^43^] in H1299 and H460 cells (Fig. 5c). According to previous studies on the role of HIF-1α in angiogenesis^44^ and the antiangiogenic effects of deguelin and its derivatives^23,25,45,46^, we further examined the antiangiogenic property of NCT-50. Because vascular endothelial cell tube formation ability in Matrigel is a general feature related to angiogenesis^47^ and angiogenic factors are produced by cancer cells and endothelial or stromal cells^48^, we assessed the effects of NCT-50 on the tube formation and proliferation of vascular endothelial cells, using the conditioned medium (CM) collected from NCT-50-treated NSCLC cells. In contrast to CM from vehicle-treated H1299 and H460 cells, exposure of HUVECs to CM from NCT-50-treated cells resulted in decreased proliferation (Fig. 5d) and tube formation (Fig. 5e) of HUVECs, indicating that NCT-50 inhibits the production of angiogenic factors by NSCLC cells. Moreover, NCT-50 exhibited improved suppressive effects on HIF-1α expression compared with the known Hsp90 inhibitors, including ganetespib and PU-H71 (Fig. 5f). These findings suggested that Hsp90 function was effectively suppressed by treatment with NCT-50.

### NCT-50 inhibits Hsp90 function by binding to the C-terminal ATP binding pocket of Hsp90

**Figure 6:**
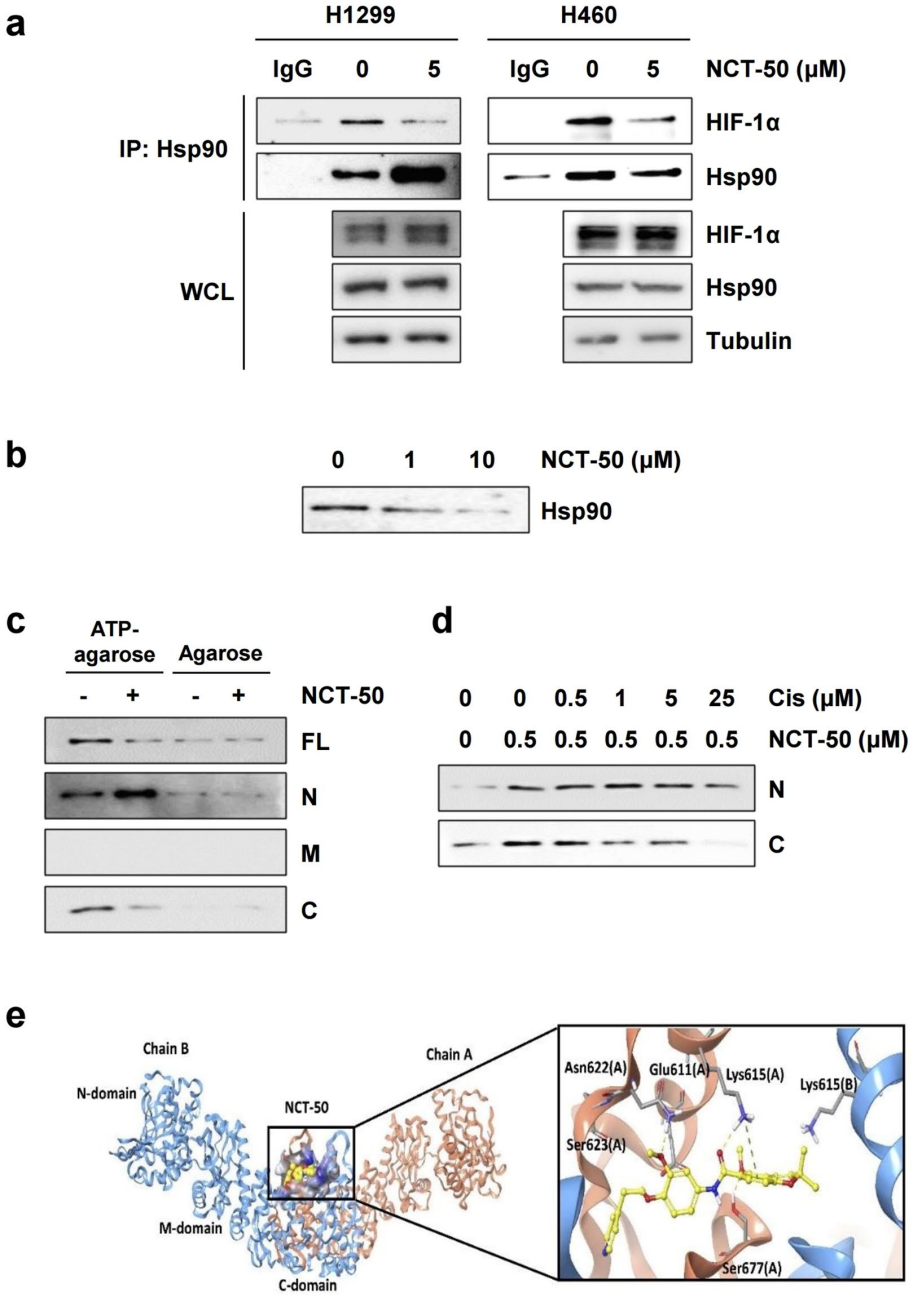
NCT-50-mediated inhibition of Hsp90 function by binding to the ATP binding pocket in the C-terminal domain of Hsp90. (**a**) H1299 and H460 cells were treated with NCT-50 (5 μM) for 1 h, followed by hypoxic incubation for 4 h. Total cell lysates were prepared and immunoprecipitated with anti-Hsp90 antibodies. The interaction between HIF-1α and Hsp90 was analyzed by Western blot analysis. (**b**) Recombinant Hsp90 protein was incubated with ATP-agarose in the presence or absence of NCT-50. The protein bound to the ATP-agarose beads was determined by Western blot analysis. (**c**) The binding of NCT-50 (5 μM) to full-length (FL) and truncated domains (N: N-terminal; M: middle; C: C-terminal) of recombinant Hsp90 proteins was determined by pull-down assay using ATP-agarose beads. Agarose beads without ATP were used to determine specific binding of Hsp90 proteins to ATP. (**d**) Competition between NCT-50 and cisplatin for the binding to the N and C domains of Hsp90 was determined by pull-down assay using biotinylated NCT-50. (**e**) *Left*. Binding site for NCT-50 in the dimerization interface of open state hHsp90. Chain A is rendered in orange ribbon, and chain B is blue ribbon. The active site is shown as electrostatic property surface map. Red, blue, and white colored regions correspond to negatively charged, positively charged, and neutral areas, respectively. *Right*. Docked pose of NCT-50 (carbon in yellow). Key amino acid residues within the binding site are rendered in grey capped stick. Hydrogen bonding interactions are depicted as yellow dashed lines and pi-cation interaction is depicted as green dashed lines. A and B indicate chain names.

We investigated the inhibitory effect of NCT-50 on Hsp90 function. Because the interaction of Hsp90 with client proteins is essential for its chaperone function^49^, we examined the effect of NCT-50 on the interaction between Hsp90 and HIF-1α. Under hypoxic conditions, the physical interaction between Hsp90 and HIF-1α was clearly reduced in H1299 and H460 cells after treatment with NCT-50 for 1 hour, when HIF-1α and Hsp90 expression was minimally affected (Fig. 6a). These findings indicated that NCT-50 biologically induce the dissociation of HIF-1α with Hsp90. Hsp90 has been proposed to possesses the ATP-binding pockets in the N-terminal and C-terminal domains^2,25,50^. To obtain evidence for NCT-50 binding to the ATP-binding pockets of Hsp90, we analyzed the effects of NCT-50 on the binding capacity of recombinant Hsp90 protein to ATP-agarose. We found that the ATP interaction with full length (FL) Hsp90 protein was suppressed by treatment with NCT-50 in a dose dependent manner (Fig. 6b). Subsequent analysis with truncated N-terminal (N) and C-terminal (C) domains revealed that ATP binding to the C domain, but not to the N domain, was markedly affected by NCT-50 treatment (Fig. 6c). To ensure the binding of NCT-50 to the C-terminal ATP-binding site of Hsp90, we performed a competition assay using biotinylated NCT-50 and a known C-terminal Hsp90 inhibitor cisplatin. Preincubation with cisplatin gradually suppressed the binding of biotinylated NCT-50 to the C-terminal domain of Hsp90 in a concentration-dependent manner (Fig. 6d). These results collectively suggest the potential of NCT-50 as a C-terminal Hsp90 inhibitor

We further conducted molecular docking analysis using the Surflex-Dox program to evaluate the binding capability of NCT-50. Previously published Hsp90 homology model structure^23,25^ was used as a receptor for docking in this study. As shown in Fig. 6d, NCT-50 fits well into the C-terminal ATP-binding cavity, locating at central region of dimerization interface of Hsp90 homodimer^51^. The oxygen atom of acylamino group forms a hydrogen bond with the side-chain of Lys615 in chain A, and the oxygen in the methoxy group forms additional hydrogen bond with the side-chain of Ser677 (A). In addition, benzopyran ring of NCT-50 engages in a cation-π interaction with the side-chain amine of Lys615. These two residues (Lys615 and Ser677) were confirmed as key residues for ATP binding in Hsp90 C-terminal domain by ATP-agarose binding assay in our previous study^25^. The pyridine and benzene fragment locate in a polar region and oxygen in the methoxy group in the benzene ring forms a hydrogen bond with side chain of Asn622. Overall, our docking model suggests that NCT-50 can efficiently bind to the C-terminal ATP binding site, stabilizing the open conformation of Hsp90 homodimer, which leads to the inhibition of Hsp90.

## Discussion

In this study, we demonstrate the potential of NCT-50 as an Hsp90-targeting anticancer agent with reduced toxicity. NCT-50 significantly inhibited the viability, colony-forming ability, and proangiogenic effect of NSCLC cells. NCT-50 induced the apoptotic cell death of NSCLC cells, but the viability of normal cells was minimally affected by NCT-50 treatment. Moreover, NCT-50 displayed comparable inhibitory effects on the survival and colony formation of NSCLC cells with acquired chemoresistance. Mechanistically, NCT-50 effectively disrupted Hsp90 function by directly binding to the C-terminal ATP-binding pocket of Hsp90, leading to downregulation of the expression and activity of several Hsp90 client proteins including HIF-1α. These data suggest that NCT-50 may have potential as an anticancer drug targeting Hsp90.

An ATPase-containing molecular chaperone, Hsp90 plays an important role in the maintenance and stabilization of numerous client proteins in response to heat shock or other stresses^1,2^. Cancer cells easily undergo proteotoxic pressure due, in part, to the accumulation of mutated molecules that could lead to cell lethality^52^. Numerous Hsp90 client proteins are often mutated or overexpressed in several types of human cancer, including NSCLC^2,53^, and these proteins mediate cancer cell proliferation, survival, angiogenesis, invasion, metastasis, and resistance to conventional or targeted anticancer drugs, such as erlotinib, trastuzumab, paclitaxel, and cisplatin^1,2,54–57^. Hence, targeting Hsp90 would be an effective way to treat cancer and overcome anticancer drug resistance. Although several clinical trials evaluating the effectiveness of Hsp90 inhibitors in lung cancer treatment are ongoing^58^, the main issues with Hsp90 inhibitors are undesirable side effects, poor water solubility, and toxicity^1^. Therefore, it is necessary to develop a novel anticancer Hsp90 inhibitor with additional chemical entities or mechanisms of action.

Previously, we demonstrated the potential of a naturally occurring rotenoid deguelin as a potent anticancer agent targeting Hsp90 through direct interaction with the ATP-binding pocket of Hsp90^18^. However, the potential neurotoxicity of deguelin due to its inhibitory effect on NADH dehydrogenase^19^ raises concern for its use in cancer patients. Hence, we have attempted to synthesize deguelin analogs to develop novel Hsp90 inhibitors^16,23–27^. According to the similar structural and mechanistic features between deguelin and novobiocin, here we synthesized a novobiocin-deguelin analog NCT-50 and demonstrated its potential to suppress NSCLC cell viability and proangiogenic ability by inhibiting Hsp90 function. We observed some features of NCT-50 that identify it as a potential hit or chemical lead for the development of anticancer Hsp90 inhibitor. NCT-50 displayed significant cytotoxic and proapoptotic effects on NSCLC cells without significantly affecting the growth of normal cells derived from lung epithelium, retinal pigment epithelium, vascular endothelium and hippocampus. The potency of the inhibitory effect of NCT-50 on the viability of NSCLC cells and Hsp90 function were comparable or superior to that of previously developed deguelin analogs, novobiocin and its analogs, and Hsp90 inhibitors in clinical trials^22–24,26,28,30^, emphasizing the potential of NCT-50 as a novel and efficacious Hsp90 inhibitor. In addition, considering liver and ocular toxicities are drawbacks of the currently available Hsp90 inhibitors^58^, the reduced toxicity of NCT-50 *in vitro* and *in vivo* compared with known Hsp90 inhibitors or deguelin appears to be a clinically favorable feature. In addition, NCT-50 significantly suppressed proangiogenic ability of NSCLC cells. Because angiogenesis is crucial for tumor growth and metastasis^59^, the antiangiogenic effect of NCT-50 may disrupt primary tumor growth and lower metastatic burden. Moreover, consistent with previous reports suggesting an association of Hsp90 with anticancer drug resistance and overcoming the resistance to chemo- or targeted anticancer therapies by using Hsp90 inhibitors^54–57^, NCT-50 was effective in both chemo-naïve and chemoresistant NSCLC cells.. Although additional studies such as animal experiments should be performed to evaluate the effectiveness and toxicity of NCT-50, either alone or in combination with chemotherapy, our results suggest the potential utility of NCT-50 to overcome resistance to paclitaxel, cisplatin, or pemetrexed, which are generally used to treat patients with NSCLC^60,61^.

Our study reveals that Hsp90 is a molecular target of NCT-50 for its anticancer and antiangiogenic effects. Consistent with the ability of deguelin analogs and novobiocin to interact with the C-terminal ATP binding pocket of Hsp90^23,25,62^, NCT-50 can interact with the C-terminal ATP binding pocket of Hsp90. Importantly, the acylamino moiety and the benzopyran ring of NCT-50 are crucial for the interaction with Lys615 and Ser677 residues of Hsp90, key residues for ATP binding in the C-terminal ATP binding pocket of Hsp90^25^. These moieties were previously defined as an important pharmacophore for the interaction of deguelin with Hsp90^18^ and the antiproliferative effect of novobiocin^28^. Thus, together with previous findings, our study provides key structural backbone for the development of C-terminal Hsp90 inhibitors.

In summary, the present study demonstrates that NCT-50 exhibits significant cytotoxic and antiangiogenic activities in NSCLC cells by suppressing Hsp90 function through directly binding to the C-terminal ATP-binding pocket of Hsp90, suggesting that NCT-50 can be considered a novel hit compound to further develop as an anticancer Hsp90 inhibitors. Further studies are warranted to investigate the effectiveness of NCT-50 in additional preclinical and clinical settings.

## Methods

### Reagents

Antibodies against EGFR, Akt, MEK1/2 and tubulin were purchased from Cell Signaling Technology (Danvers, MA, USA). Antibodies against cleaved PARP and HIF-1α were purchased from BD Biosciences (San Jose, CA, USA). Antibodies against IGF-1R and actin were purchased from Santa Cruz Biotechnology (Santa Cruz, CA, USA). HRP-conjugated anti-rabbit and anti-goat secondary antibodies were purchased from GeneTex (Irvine, CA, USA). An anti-Hsp90 antibody was purchased from Enzo Life Science (Farmingdale, NY, USA). Ni-NTA agarose was purchased from Invitrogen (Carlsbad, CA, USA). ATP-agarose was acquired from Innova Biosciences (Cambridge, UK). The first-strand cDNA synthesis kit was purchased from Dakara Korea Biomedical, Inc. (Seoul, Republic of Korea). 3-(4,5-Dimethylthiazol-2-yl)-2,5-diphenyltetrazolium bromide (MTT), propidium iodide (PI), crystal violet, and other chemicals were purchased from Sigma-Aldrich (St. Louis, MO, USA) unless otherwise specified.

### Cell culture

H1299, H460, A549, H226B, and BEAS-2B cells were purchased from the American Type Culture Collection (ATCC, Manassas, VA, USA) or kindly provided by Dr. John V. Heymach (The University of Texas M. D. Anderson Cancer Center, Houston, TX, USA). Human umbilical vein endothelial cells (HUVECs) were purchased from Invitrogen (Carlsbad, CA, USA). Human retinal pigment epithelial (RPE) cells were kindly provided by Dr. Jeong Hun Kim (College of Medicine, Seoul National University, Seoul, Republic of Korea). Murine hippocampal neuronal cell line HT-22 was kindly provided by Dr. Dong Gyu Jo (College of Pharmacy, Sungkyunkwan University, Suwon, Republic of Korea). Human bronchial epithelial (HBE) cells were kindly provided by Dr. John Minna (The University of Texas Southwestern Medical Center, Dallas, TX, USA). H1299/PmR, H1299/CsR, and H226B/PcR cells were generated by continuous exposure to increasing concentrations of pemetrexed (for PmR), cisplatin (for CsR), or paclitaxel (for PcR) for more than 6 months^25^. All NSCLC cell lines were authenticated and validated. NSCLC cells were cultured in RPMI 1640 medium supplemented with 10% fetal bovine serum (FBS) and antibiotics (all from Welgene Inc., Gyeongssan-si, Republic of Korea). RPE and HT-22 cells were cultured in DMEM (Welgene) supplemented with 10% FBS and antibiotics. HBE and BEAS-2B cells were cultured in Keratinocyte-SFM (Invitrogen, Grand Island, NY, USA) supplemented with 5 ng/ml recombinant epidermal growth factor (EGF), 50 μg/ml bovine pituitary extracts, and antibiotics. HUVECs were grown in VascuLife basal medium supplemented with VascuLife VEGF life factors (Lifeline Cell Technology, Frederick, MD, USA). Cells were incubated at 37 °C with 5% CO_2_ in a humidified atmosphere.

### MTT assay

Cells [2 × 10^3^ cells/well (for NSCLC and HT-22 cells) or 3 × 10^3^ cells/well (for HBE, BEAS-2B, RPE, and HUVEC) in 96-well plates] were treated with increasing concentrations of NCT-50, deguelin, ganetespib, or PU-H71 (0.1, 1, and 10 μM) for 2 or 3 days. After incubation, the cells were incubated with MTT solution (final concentration of 200 μg/ml) and incubated for 2 h at 37 °C. The formazan products were dissolved in DMSO, and the absorbance was measured at 570 nm. The data are presented as a percentage of the control group.

### Anchorage-dependent and anchorage-independent colony formation

For the anchorage-dependent colony formation assay, cells were plated at a density of 300 cells/well in six-well plates and were treated with increasing concentrations of NCT-50 for 2 weeks. Colonies were fixed with 100% methanol, stained with 0.02% crystal violet solution at room temperature, photographed, and counted.

For the anchorage-independent colony formation assay, cells were mixed with sterile 1% agar solution (final concentration of 0.4%) and then poured onto the 1% base agar in 12- or 24-well plates. After solidification, NCT-50 diluted in complete medium was added to the agar and incubated for 2 weeks. Colonies were stained with the MTT solution, photographed, and counted.

### Cell cycle analysis

Cells were treated with 0, 1, and 5 μM NCT-50 for three days. All adherent or floating cells were collected and washed with PBS. Cells were fixed with 100% methanol and stained with 50 μg/ml PI solution containing 50 μg/ml RNase A for 30 min at room temperature. Changes in the cell cycle distribution by NCT-50 treatment were determined by flow cytometry using a FACSCalibur® flow cytometer (BD Biosciences, San Jose, CA, USA).

### Annexin V/PI double staining

Cells were plated into a 6-well plate at a density of 1.2 × 10^5^ cells (for H1299 cells) or 1.5 × 10^5^ cells (for H460) and incubated for 24 h. Cells were treated with vehicle or NCT-50 (1 or 5 μM) for additional two days. Cells were harvested by trypsinization, stained with Annexin V-FITC or PI, and analyzed by flow cytometry according to the manufacturer’s recommended procedure (BD Biosciences).

### Tube formation assay

NSCLC cells were treated with NCT-50 for 24 h. Then, cell media were exchanged with fresh serum-free media. Conditioned media (CM) were collected 24 h after incubation. A 96-well plate was coated with growth factor reduced Matrigel (Corning, Corning, NY, USA). Then, HUVECs were seeded at a 104 cells/well density. For CM treated groups, each CM were mixed (1:1) with VasCulife media with 0.4% FBS. After 18 h, tube formation was analyzed under a microscope.

### Nuclear morphology analysis

H1299 and H460 cells were treated with different dose of NCT-50 for 36 h. After treatment, the cells were incubated with Hoechst 33342 (Thermo Fisher Scientific, Waltham, MA, USA) diluted in fresh serum containing media (20 μM) for 30 min, then observed under fluorescent microscope to survey condensed, fragmented or degraded nuclei in each cell and counted.

### Western blot analysis

Cells were treated with increasing concentrations of NCT-50 (0, 1, and 5 μM) for 24 h. When necessary, the cells were further incubated under hypoxic conditions (1% O_2_ for 4 h). The cells were harvested with RIPA lysis buffer as described previously^18^. Equal amounts of cell lysates were resolved by 8–15% SDS-PAGE and transferred onto a PVDF membrane. Membranes were blocked with blocking buffer [3% skim milk in TBS containing 0.1% Tween-20 (TBST)] for 1 h at room temperature and incubated with primary antibodies diluted in TBST containing 3% bovine serum albumin (BSA) for overnight at 4 °C. Membranes were washed three times with TBST for 1 h at room temperature and incubated with the corresponding secondary antibodies diluted in 3% skim milk in TBST (1:5000) for 1 h at room temperature. Membranes were washed three times with TBST and visualized using an enhanced chemiluminescence (ECL) detection kit (Thermo Fisher Scientific, Waltham, MA, USA).

### Real-time PCR

Cells were treated with various concentrations of NCT-50 for 24 h and then harvested. Total RNA was prepared using an easy-BLUE total RNA extraction kit (Intron Biotechnology, Sungnam-si, Kyunggi-do, Republic of Korea) according to the manufacturer’s protocol. We performed a SYBR Green-based real-time PCR analysis according to the protocol provided by the manufacturer (Bioneer Corp., Daejeon, Republic of Korea). The primer sequences used for real-time PCR are as follows: human vascular endothelial growth factor (*VEGF*) forward, 5′-CTT GCC TTG CTG CTC TAC C-3′; human *VEGF* reverse, 5′-CAC ACA GGA TGG CTT GAA G-3′; human transforming growth factor beta 3 (*TGFB3*) forward, 5′-TCT CCC AGA TCG GTG ACA GT-3′; human *TGFB3* reverse, 5′-TGT CGG AAG TCA ATG TAG AG-3′; human platelet derived growth factor (*PDGFB*) forward, 5′- TTT CTC ACC TGG ACA GG CG-3′; human *PDGFB* reverse, 5′-GAA GGA GCC TGG GTT CCC T-3′; human *DDIT3* forward, 5′- GGA GCA TCA GTC CCC CAC TT-3; human *DDIT3* reverse, 5′- TGT GGG ATT GAG GGT CAC ATC-3; human *PPP1R15A* forward, 5′- CCC AGA AAC CCC TAC TCA TGA TC -3; human *PPP1R15A* reverse, 5′- GCC CAG ACA GCC AGG AAA T -3; human *TRIB3* forward, 5′- TGC GTG ATC TCA AGC TGT GT -3; human *TRIB3* reverse, 5′- GCT TGT CCC ACA GGG AAT CA -3; human *ACTB* forward, 5′- GCG AGA AGA TGA CCC AGA TC-3′; human *ACTB* reverse, 5′-GAT CCA CAT CTG CTG GAA-3′. The thermocycler conditions for real-time PCR were as follows: pre-incubation at 95 °C for 5 min, 30–40 cycles of 95 °C for 10 sec, 60 °C for 10 sec, and 72 °C for 10 sec, and melting curve analysis was performed to determine reaction specificity. Relative quantification of mRNA expression was performed by the comparative CT (cycle threshold) method as described previously^63^.

### NADH dehydrogenase activity assay

NADH dehydrogenase activity assay was performed according to the previously reports with some modifications^64,65^. Confluent H460 cells in 100 mm culture dishes were washed twice with ice-cold PBS and harvested by scraping in PBS. After centrifugation, cell pellets were suspended in 1 ml ice-cold 10 mM Tris (pH 7.4) and mechanistically disrupted by sonication using Vibra-cell ultrasonic processor (Sonics & Materials Inc., Newtown, CT, USA). After adding 0.2 ml ice-cold 1.5 M sucrose, the suspension was centrifuged at 600 × g for 10 min at 4 °C. Supernatants were further centrifuged at 14,000 × g for 10 min at 4 °C.Pellets were suspended in 100 μl 10 mM Tris (pH 7.4). Protein concentration was determined using BCA assay kit (Thermo Fisher Scientific). A mitochondria enriched fraction (20 μl, containing 20 μg of protein) was incubated with 960 μl of the incubation mixture containing 25 mM potassium phosphate (pH 7.8), 3.5 g/L fatty acid-free bovine serum albumin (BSA), 60 μM 2,6-dichloroindophenol, 70 μM decylubiquinone, and 1 μM antimycin A for 1 min at room temperature. The mixture was treated with vehicle or test compounds (5 μM NCT-50 or deguelin). After measuring the absorbance at 600 nm, 20 μl of 10 mM NADH solution was added, and the absorbance was monitored at 1 min intervals for 10 min. Enzyme activity of the test group was calculated according to the following formula and expressed as a percentage of the vehicle-treated control group; enzyme activity (nmol/min/mg) = (Δ Absorbance/min × 1,000)/[(extinction coefficient of DCIP × volume of sample used in 1 ml) × (sample protein concentration in mg/ml)]^65^. Extinction coefficient of DCIP was 19.1 mM^−1^cm^−1^ ^64,65^.

### Immunoprecipitation and pull-down assay

Immunoprecipitation, the purification of Hsp90 proteins, and a pull-down assay to determine the competitive binding to the ATP-binding pocket using ATP-agarose were performed according to our previous report^18^.

### *In vivo* toxicity test

Animal experiments were performed according to protocols approved by the Seoul National University Institutional Animal Care and Use Committee. Mice were fed standard mouse chow and water *ad libitum* and housed in temperature- and humidity-controlled facilities with a 12-h light/12-h dark cycle. 6-weeks-old FVB mice were treated with vehicle (10% DMSO in corn oil) or test compounds (deguelin or NCT-50, 4 mg/kg) twice a day for 7 consecutive days. Body weight changes were monitored during the treatment. *In vivo* toxicity test was perform as described previously^66^. Briefly, blood was collected from euthanized mice by cardiac puncture. Serum was collected by centrifugation at 3,000 rpm for 10 min at 4 °C. The level of GOT, GPT, and BUN in the serum was analyzed using a veterinary hematology analyzer (Fuji DRI-Chem 3500 s, Fujifilm, Tokyo, Japan) according to the manufacturer’s recommended procedure. The histopathological changes in liver, lung, brain, and kidney were evaluated by using H&E-stained section of the tissues.

### Immunofluorescence

Frozen sections of the mouse midbrain were fixed with 4% paraformaldehyde for 30 min at room temperature. After washing twice with PBS for 5 min each, sections were permeabilized with 0.2% Triton X-100 solution for 15 min at room temperature. Sections were washed three times with PBS for 5 min each. Sections were blocked with blocking buffer (3% BSA solution containing 10% normal donkey serum) for 1 h at room temperature, and then incubated with anti-tyrosine hydroxylase primary antibody (EMD Millipore, Burlington, MA, USA; 1:200 dilution) for overnight at 4 °C. Slides were washed three times with PBS for 10 min each at room temperature. Slides were further incubated with corresponding Alexa 488-conjugated secondary antibody (Thermo Fisher Scientific; 1:500 dilution) for 1 h at room temperature. Slides were washed six times with PBS for 10 min each and then mounted with mounting medium with DAPI. Images were acquired with a confocal microscope (LSM 700; Carl Zeiss Microscopy, Jena, Germany).

### Docking modeling

All performances of computational works were carried out on the Tripos Sybyl-X 2.1 (Tripos Inc, St Louis, MO, USA) molecular modeling package.

#### Preparation of ligands

The ligands were prepared as Mol2 format using sketch modules embedded in Sybyl. Gasteiger-Hückel charges were assigned to all ligands atoms. Each ligand was energy-minimized using a standard Tripos force field with convergence to maximum derivatives of 0.001 kcal mol-1.Å-1.

#### Homology modeling of open conformation of hHsp90 homodimer

We conducted the homology modeling of full-length of human Hsp90α by using ORCHESTRAR module. It was reported that the function of Hsp90 C-terminal inhibitors was related with binding to the open form of homodimer Hsp90 structure^67^. Based on this information, we built up an open conformation of human Hsp90 dimer based on the extended SAXS model of E. coli Hsp90 homodimer including N-, middle and C-terminal domain. The template structure was retrieved from Agard’ lab website (http://www.msg.ucsf.edu/agard, PDB id: hsp90). The sequence identity of Hsp90 between E-coli and human is 42.9%. First, alignment file was defined using complete partial model. Then, based on this alignment file, structurally conserved regions (SCRs) were built automatically, and structurally variable region was identified. SCRs including loops were optimized by loop-search option. Side chains of amino acids were fixed with set side chain option. To remove the bad contacts in the protein structure, the brief energy-minimization of model was performed with assigning Kollman all charges to all the atoms of protein and convergence to maximum derivatives of 0.05 kcal mol-1.Å-1.

#### Molecular docking

The compound NCT-50 was docked into the active site of hHsp90 homology model structure using Surflex-Dock algorithm. The active site was defined by generating a protomol based on the ATP- binding pocket of the C-terminal domain. The protomol was built using the hydrogen-containing protein mol2 file with optimized parameters (threshold factor of 0.4 Å and bloat of 1 Å). Docking was performed with setting default parameters and 20 maximum ligand conformers to generate. Binding affinity of each docking pose of ligand was calculated by Surflex-Dock score (-log Kd). Based on the docking score and visual inspection, top-ranked docking model was selected.

### Statistical analysis

The data are presented as the mean ± SD. The statistical significance was determined using a two-sided Student’s *t*-test using GraphPad Prism 5 (GraphPad Software Inc., La Jolla, CA, USA). The IC_50_ values were determined by non-linear regression analysis using Graphpad Prism 5 (GraphPad Software, Inc., La Jolla, CA, USA). A *P* value less than 0.05 was statistically significant.

### Synthesis of NCT-50

#### General Methods

All chemical reagents were commercially available. Melting points were determined on a melting point Buchi B-540 apparatus. Silica gel column chromatography was performed on silica gel 60, 230–400 mesh, Merck. ^1^H-NMR analyses were recorded on a JEOL JNM-LA 300 at 300 MHz. Chemical shifts are reported in ppm units with Me_4_Si as a reference standard. Mass spectra were recorded on a VG Trio-2 GC−MS instrument and a 6460 Triple Quad LC−MS instrument. All final compounds were assessed for purity by high performance liquid chromatography (HPLC) on Agilent 1120 Compact LC (G4288A) system via the following conditions: column: Agilent TC-C18 column (4.6 mm × 250 mm, 5 μm). Mobile phase A: 0.1% TFA water. Mobile phase B: 0.1% TFA MeOH (50:50, v/v). Wavelength: 254 nM. Flow: 0.50 mL/min.

#### 2-(Pyridin-4-yl)ethan-1-ol (2)

To a cooled solution of ethyl 2-(pyridin-4-yl)acetate (1, 200 mg, 1.21 mmol) in THF (6 mL) at 0 °C, lithium aluminium hydride (92 mg, 2.42 mmol) was added portionwise. The reaction mixture was stirred at room temperature for 1 h and monitored by TLC. Upon completion, the reaction was terminated by adding H_2_O. The organic layer was extracted with CH_2_Cl_2_, dried over MgSO_4_ and concentrated *in vacuo*. The residue was purified by column chromatography (CH_2_Cl_2_/CH_3_OH, 20:1) to give 2 as a yellow oil (110 mg, 74%). ^1^H-NMR (300 MHz, CDCl_3_) δ 8.47 (dd, 2 H, *J* = 4.38, 1.65 Hz, *H*_2_, *H*_6_-pyridine), 7.15 (dd, 2 H, *J* = 4.38, 1.65 Hz, *H*_3_, *H*_5_-pyridine), 3.89 (t, 2 H, *J* = 6.42 Hz, -CH_2_C*H*_2_OH), 2.84 (t, 2 H, *J* = 6.42 Hz, -C*H*_2_CH_2_OH).

#### 4-(2-(2-Methoxy-4-nitrophenoxy)ethyl)pyridine (3)

To a cooled solution of 2 (110 mg, 0.89 mmol), 4-nitroguaiacol (227.26 mg, 1.34 mmol) and triphenylphosphine (352.6 mg, 1.34 mmol) in CH_2_Cl_2_ (20 mL) at 0 °C, diethyl azodicarboxylate (0.6 mL, 1.34 mmol, 40% solution in toluene) was added dropwise. The reaction mixture was stirred overnight at ambient temperature. Upon completion, the reaction was terminated by adding H_2_O. The organic layer was extracted with CH_2_Cl_2_, dried over MgSO_4_ and concentrated *in vacuo*. The residue was purified by column chromatography (CH_2_Cl_2_/CH_3_OH, 20:1) to give 3 as a yellow solid (66 mg, 26%). ^1^H-NMR (300 MHz, CDCl_3_) δ 8.53 (dd, 2 H, *J* = 4.38, 1.65 Hz, *H*_2_,*H*_6_-pyridine), 7.86 (dd, 1 H, *J* = 8.97, 2.55 Hz, *H*_5_-benzene), 7.73 (d, 1 H, *J* = 2.55 Hz, *H*_3_-benzene), 7.22 (dd, 2 H, *J* = 4.38, 1.65 Hz, *H*_*3*_,*H*_5_-pyridine), 6.86 (d, 1 H, *J* = 8.97 Hz, *H*_6_-benzene), 4.30 (t, 2 H, *J* = 6.78 Hz, -CH_2_C*H*_2_O-), 3.92 (s, 3 H, C*H*_3_O-), 3.17 (t, 2 H, *J* = 6.78 Hz, -C*H*_2_CH_2_O-).

#### 3-Methoxy-4-(2-(pyridin-4-yl)ethoxy)aniline (4)

A mixture of 3 (65.96 mg, 0.24 mmol) and 10% Pd/C (6 mg) in CH_3_OH (5 mL) was hydrogenated under a balloon of hydrogen for 2 h. The reaction mixture was filtered over Celite and the combined filtrate was concentrated *in vacuo*. The residue was purified by column chromatography (CH_2_Cl_2_/CH_3_OH, 10:1) to give 4 as a brown solid (49.1 mg, 84%). ^1^H-NMR (300 MHz, CDCl_3_) δ 8.50 (dd, 2 H, *J* = 4.41, 1.65 Hz, *H*_2_, *H*_6_-pyridine), 7.21 (dd, 2 H, *J* = 4.41, 1.65 Hz, *H*_3_, *H*_5_-pyridine), 6.67 (d, 1 H, *J* = 8.43 Hz, *H*_5_-benzene), 6.27 (d, 1 H, *J* = 2.55 Hz, *H*_2_-benzene), 6.17 (dd, 1 H, *J* = 8.43, *J* = 2.58 Hz, *H*_6_-benzene), 4.13 (t, 2 H, *J* = 6.96 Hz, -CH_2_C*H*_2_O-), 3.77 (s, 3 H, C*H*_3_O-), 3.49 (*b*r, 2 H, -N*H*_2_), 3.05 (t, 2 H, *J* = 6.78 Hz, -C*H*_2_CH_2_O-).

#### 5-Hydroxy-2,2-dimethyl-2H-chromene-6-carbaldehyde (6)

To a stirred solution of 2,4-dihydroxybenzaldehye (**5**, 5.53 g, 40 mmol) in pyridine (50 ml), 3-methylbutenal (2 mL, 20 mmol) was added and then the reaction mixture was refluxed for 15 h. Upon completion, the reaction was allowed to warm to ambient temperature and quenched with 1 N HCl. The organic layer was extracted with EtOAc, dried over MgSO_4_, concentrated *in vacuo*. The residue was purified by column chromatography (hexanes/EtOAc, 1:1) to give **6** as yellow solid (1.35 g, 32%). ^1^H-NMR (300 MHz, CDCl_3_) δ 11.62 (s, 1 H, O*H*), 9.63 (s, 1 H, -C*H*O), 7.26 (d, 1 H, *J* = 8.43 Hz, *H*_7_-chromene), 6.66 (d, 1 H, *J* = 10.05 Hz, *H*_4_-chromene), 6.40 (dd, 1 H *J* = 8.61, 0.72 Hz, *H*_8_-chromene), 5.58 (d, 1 H, 10.08 Hz, *H*_3_-chromene), 1.49 (s, 6 H, -(C*H*_3_)_2_).

#### 5-Methoxy-2,2-dimethyl-2H-chromene-6-carbaldehyde (7)

To a stirred solution of 6 (1.35 g, 6.6 mmol) in DMF (50 mL), potassium carbonate (3.69 g, 26.7 mmol) followed by iodomethane (1.24 mL, 19.9 mmol) was added and then the reaction mixture was stirred at 70 °C for 3 h. Upon completion, the reaction was allowed to warm to ambient temperature and quenched with H_2_O and extracted with EtOAc, dried over MgSO_4_ and concentrated *in vacuo*. The residue was purified by column chromatography (hexanes/EtOAc, 1:1) to give **7** as a brown oil (1.05 g, 72%). ^1^H-NMR (300 MHz, CDCl_3_) δ 10.15 (s, 1 H, -C*H*O), 7.63 (d, 1 H, *J* = 8.58 Hz, *H*_7_-chromene), 6.62 (d, 1 H, *J* = 8.61 Hz, *H*_4_-chromene), 6.57 (d, 1 H, *J* = 10.08 Hz, *H*_8_-chromene), 5.67 (d, 1 H, *J* = 10.08, *H*_3_-chromene), 3.88 (s, 3 H, C*H*_3_O-), 1.44 (s, 6 H, -(C*H*_3_)_2_).

#### 5-Methoxy-2,2-dimethyl-2H-chromene-6-carboxylic acid (8)

To a stirred solution of 7 (1.05 g, 4.81 mmol), NaH_2_PO_4_ (114 mg, 0.95 mmol), 30% H_2_O_2_ (1.82 mL, 4.81 mmol) in Acetonitrile (50 mL) at room temperature, NaClO_2_ (652.8 mg, 7.22 mmol) was added and the reaction mixture was stirred at room temperature for 2 h. Upon completion, the reaction mixture was extracted with EtOAc, dried over MgSO_4_ and concentrated *in vacuo*. The residue was purified by column chromatography (hexanes/EtOAc, 1:1) to give 8 as yellow solid (710.8 mg, 63%). ^1^H-NMR (300 MHz, CDCl_3_) δ 7.89 (d, 1 H, *J* = 8.79 Hz, *H*_7_-chromene), 6.68 (dd, 1 H, *J* = 8.79, 0.72 Hz, *H*_4_-chromene), 6.52 (dd, 1 H, *J* = 10.05, 0.54 Hz, *H*_8_-chromene), 5.72 (d, 1 H, *J* = 9.87 Hz, *H*_3_-chromene), 3.93 (s, 3 H, C*H*_3_O-), 1.45 (s, 6 H, -(CH_3_)_2_).

#### 5-Methoxy-N-(3-methoxy-4-(2-(pyridin-4-yl)ethoxy)phenyl)-2,2-dimethyl-2H-chromene-6-carboxamide (9)

To a stirred solution of 4 (48.8 mg, 0.2 mmol), 1-ethyl-3-(3-dimethylaminopropyl)carbodiimide (57.5 mg, 0.3 mmol), hydroxybenzotriazole (40.6 mg, 0.3 mmol) and triethylamine (0.07 mL, 0.5 mmol) in CH_2_Cl_2_ (20 mL), 8 (47.1 mg, 0.2 mmol) in CH_2_Cl_2_ (10 mL) was added and the reaction mixture was refluxed for 15 h. Upon completion, the reaction mixture was extracted with EtOAc, dried over Mg_2_SO_4_ and concentrated *in vacuo*. The residue was purified by column chromatography (hexanes/EtOAc, 1:1) to give 9 as a brown solid (71.2 mg, 77%). Anal. HPLC 99% (R_t_ = 4.5 min), mp = 112–118 °C, ^1^H-NMR (300 MHz, CDCl_3_) δ 9.61 (s, 1 H, -CN*H*CO-), 8.51 (dd, 2 H, *J* = 4.38, 1.65 Hz, *H*_2_, *H*_6_-pyridine), 7.93 (d, 1 H, *J* = 8.79 Hz, *H*_7_-chromene), 7.62 (d, 1 H, *J* = 2.37 Hz, *H*_2_-benzene), 7.23 (dd, 2 H, *J* = 4.38, 1.65 Hz, *H*_2_, *H*_5_-pyridine), 6.89 (dd, 1 H, *J* = 8.61, 2.37 Hz, *H*_6_-benzene), 6.80 (d, 1 H, *J* = 8.43 Hz, *H*_8_-chromene), 6.70 (d, 1 H, *J* = 8.61 Hz, *H*_5_-benzene), 6.57 (d, 1 H, *J* = 9.90 Hz, *H*_4_-chromene), 5.71 (d, 1 H, *J* = 10.08 Hz, *H*_3_-chromene), 4.22 (t, 2 H, *J* = 6.78 Hz, -CH_2_C*H*_2_O-), 3.88 (s, 3 H, C*H*_3_O-), 3.58 (s, 3 H, C*H*_3_O-), 3.11 (t, 2 H, *J* = 6.78 Hz, -C*H*_2_CH_2_O-), 1.44 (s, 6 H, -(C*H*_3_)_2_). HRMS (ESI) calc. for C_27_H_28_N_2_O_5_ [M + H]^+^ 461.2071, found 461.2064.

### Ethical approval and informed consent

Animal experiments were performed according to protocols approved by the Seoul National University Institutional Animal Care and Use Committee..

## Electronic supplementary material

Supplementary information

## Data Availability

All data generated or analyzed during this study are included in this published article
