# Supplementary material for: Development of a novel Hsp90 inhibitor NCT-50 as a potential anticancer agent for the treatment of non-small cell lung cancer
Source: Sci Rep. 2018 Sep 17;8:13924. doi: 10.1038/s41598-018-32196-6 (PMC6141536; doi:10.1038/s41598-018-32196-6)
Supplement: Supplementary file 1 — Supplementary information [file 41598_2018_32196_MOESM1_ESM.pdf]

Development of a novel Hsp90 inhibitor NCT-50 as a potential anticancer agent for the treatment of non-small cell lung cancer

Seung Yeob Hyun, Huong Thuy Le, Cong-Truong Nguyen, Young-Sik Yong, Hye-Jin Boo, Ho Jin Lee, Ji-Sun Lee, Hye-Young Min, Jihyae Ann, Jie Chen, Hyun-Ju Park, Jeewoo Lee, Ho-Young Lee

Figure 3c

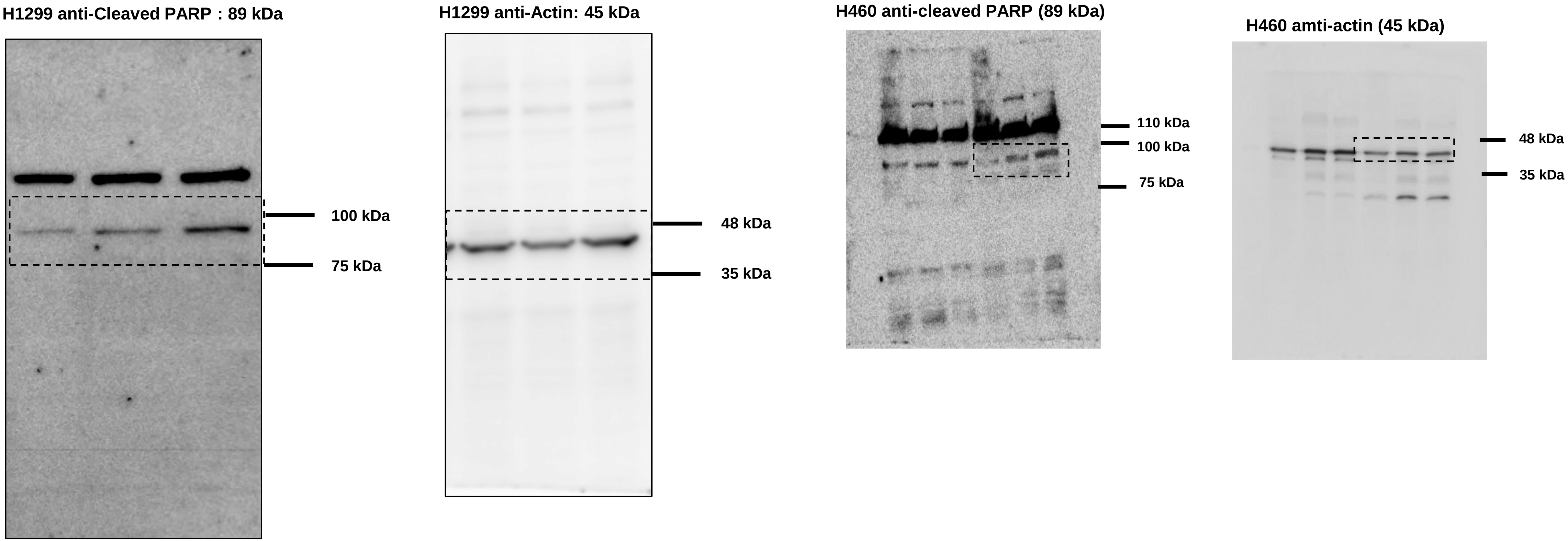

H1299 anti-Cleaved PARP : 89 kDa
100 kDa
75 kDa
H1299 anti-Actin: 45 kDa
48 kDa
35 kDa
H460 anti-cleaved PARP (89 kDa)
110 kDa
100 kDa
75 kDa
H460 amti-actin (45 kDa)
48 kDa
35 kDa

Figure 5a

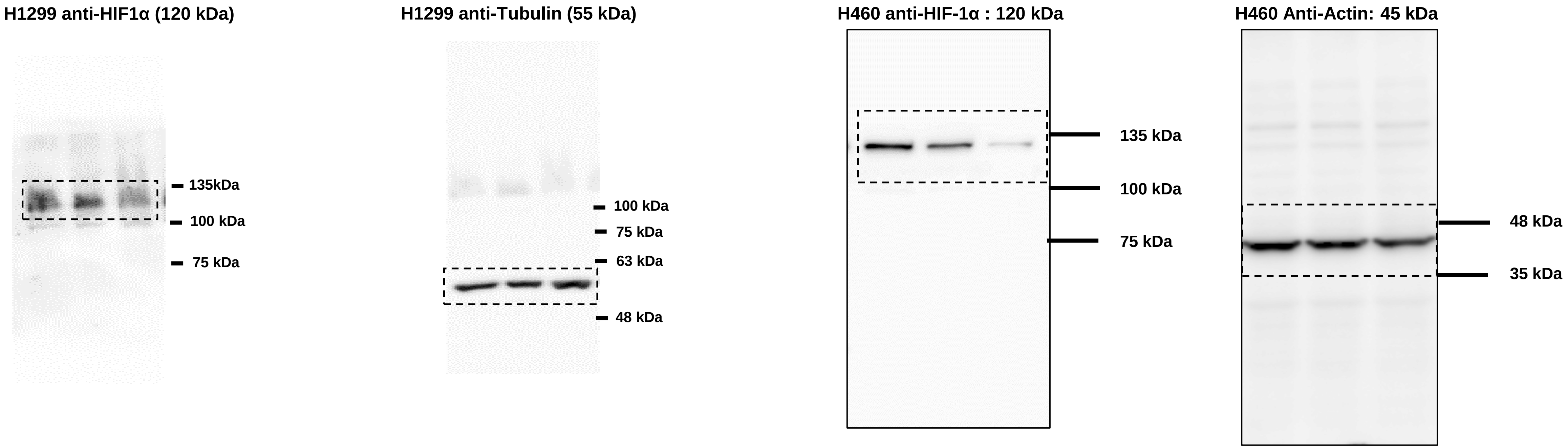

H1299 anti-HIF1α (120 kDa)
135kDa
100 kDa
75 kDa
H1299 anti-Tubulin (55 kDa)
100 kDa
75 kDa
63 kDa
48 kDa
H460 anti-HIF-1α : 120 kDa
135 kDa
100 kDa
75 kDa
H460 Anti-Actin: 45 kDa
48 kDa
35 kDa

Figure 5b

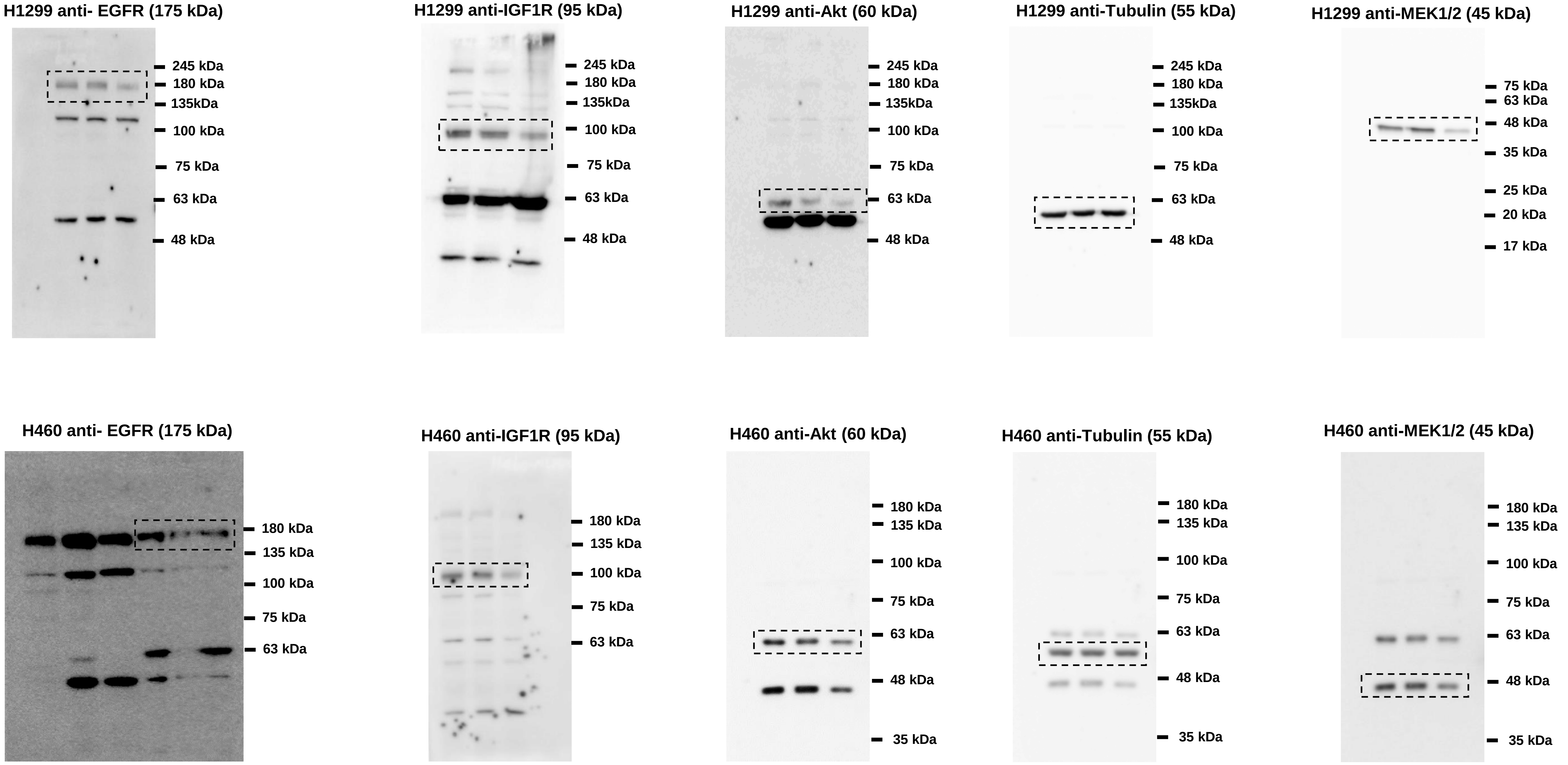
H1299 anti- EGFR (175 kDa)
245 kDa
180 kDa
135kDa
100 kDa
75 kDa
63 kDa
48 kDa
H1299 anti-IGF1R (95 kDa)
245 kDa
180 kDa
135kDa
100 kDa
75 kDa
63 kDa
48 kDa
H1299 anti-Akt (60 kDa)
245 kDa
180 kDa
135kDa
100 kDa
75 kDa
63 kDa
48 kDa
H1299 anti-Tubulin (55 kDa)
245 kDa
180 kDa
135kDa
100 kDa
75 kDa
63 kDa
48 kDa
H1299 anti-MEK1/2 (45 kDa)
75 kDa
63 kDa
48 kDa
35 kDa
25 kDa
20 kDa
17 kDa
H460 anti- EGFR (175 kDa)
180 kDa
135 kDa
100 kDa
75 kDa
63 kDa
H460 anti-IGF1R (95 kDa)
180 kDa
135 kDa
100 kDa
75 kDa
63 kDa
H460 anti-Akt (60 kDa)
180 kDa
135 kDa
100 kDa
75 kDa
63 kDa
48 kDa
35 kDa
H460 anti-Tubulin (55 kDa)
180 kDa
135 kDa
100 kDa
75 kDa
63 kDa
48 kDa
35 kDa
H460 anti-MEK1/2 (45 kDa)
180 kDa
135 kDa
100 kDa
75 kDa
63 kDa
48 kDa
35 kDa

Figure 5g

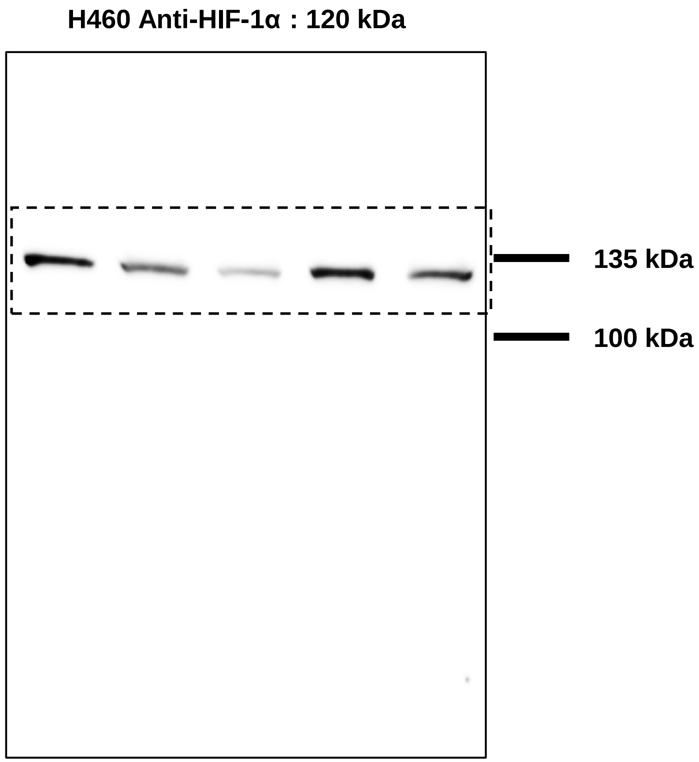
H460 Anti-HIF-1α : 120 kDa
135 kDa
100 kDa

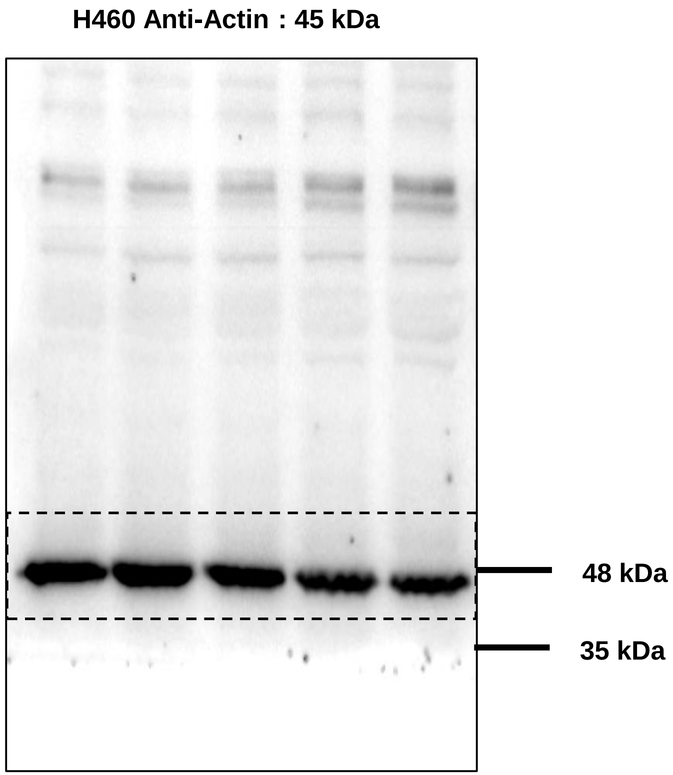
H460 Anti-Actin : 45 kDa
48 kDa
35 kDa

Figure 6a

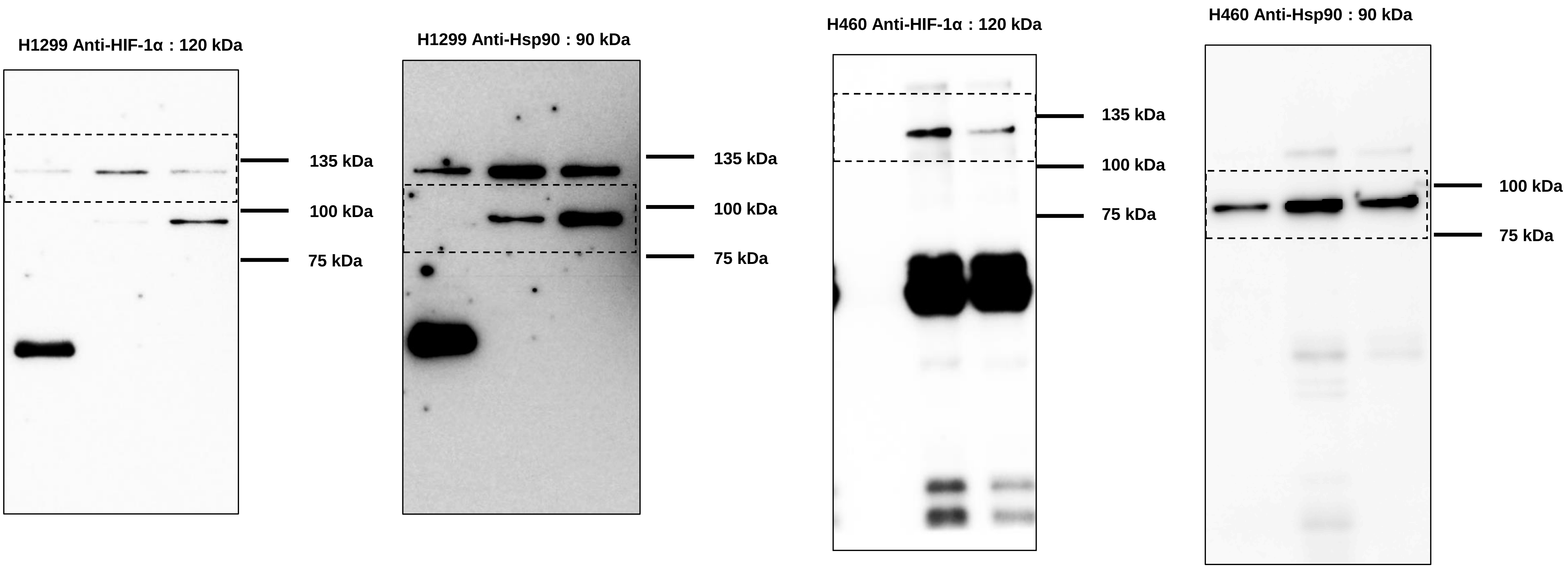
H1299 Anti-HIF-1α : 120 kDa
135 kDa
100 kDa
75 kDa
H1299 Anti-Hsp90 : 90 kDa
135 kDa
100 kDa
75 kDa
H460 Anti-HIF-1α : 120 kDa
135 kDa
100 kDa
75 kDa
H460 Anti-Hsp90 : 90 kDa
100 kDa
75 kDa

Figure 6a

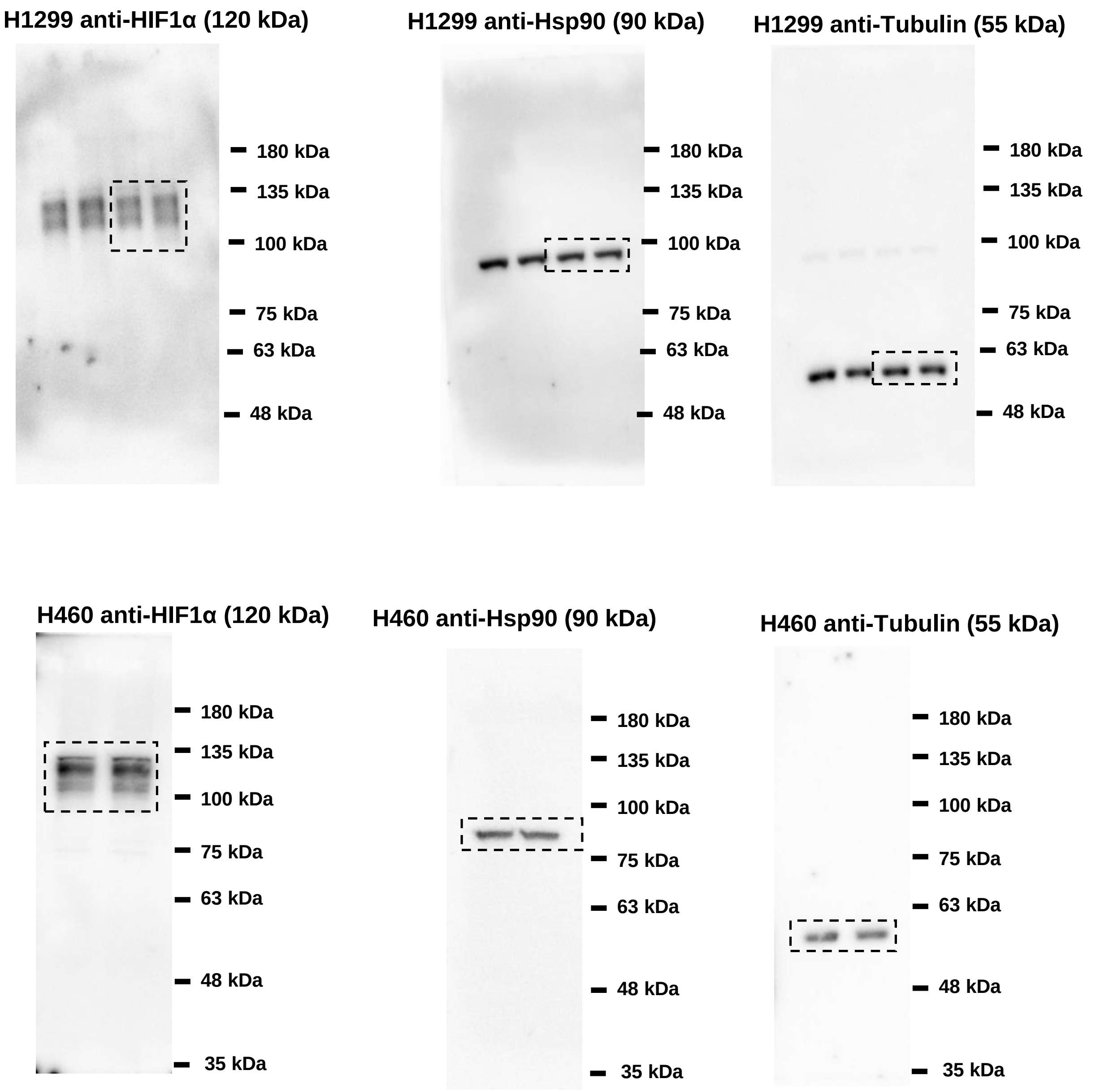
H1299 anti-HIF1α (120 kDa)
H1299 anti-Hsp90 (90 kDa)
H1299 anti-Tubulin (55 kDa)
180 kDa
135 kDa
100 kDa
75 kDa
63 kDa
48 kDa
H460 anti-HIF1α (120 kDa)
H460 anti-Hsp90 (90 kDa)
H460 anti-Tubulin (55 kDa)
180 kDa
135 kDa
100 kDa
75 kDa
63 kDa
48 kDa
35 kDa

Figure 6b

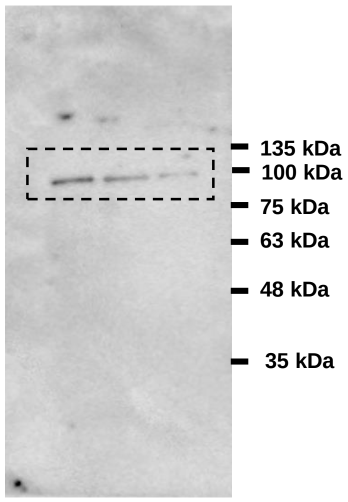

Figure 6c

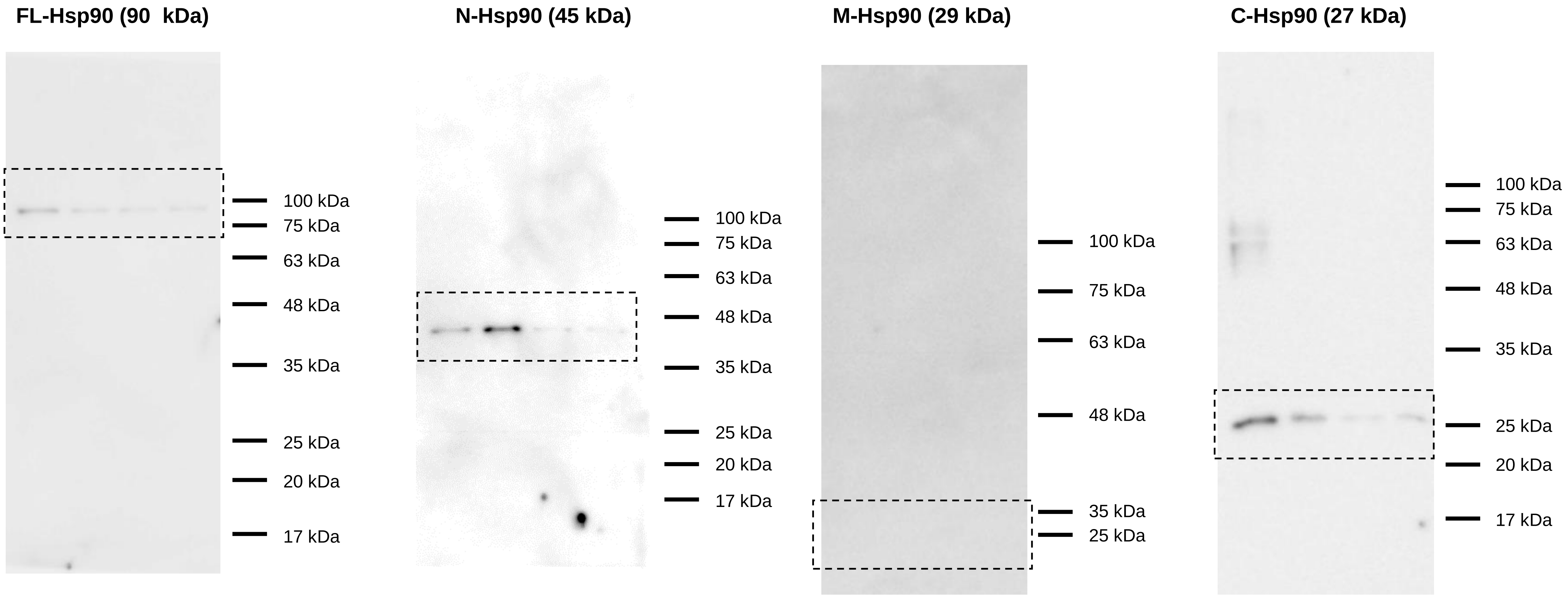
FL-Hsp90 (90 kDa)
100 kDa
75 kDa
63 kDa
48 kDa
35 kDa
25 kDa
20 kDa
17 kDa
N-Hsp90 (45 kDa)
100 kDa
75 kDa
63 kDa
48 kDa
35 kDa
25 kDa
20 kDa
17 kDa
M-Hsp90 (29 kDa)
100 kDa
75 kDa
63 kDa
48 kDa
35 kDa
25 kDa
C-Hsp90 (27 kDa)
100 kDa
75 kDa
63 kDa
48 kDa
35 kDa
25 kDa
20 kDa
17 kDa

Figure 6d

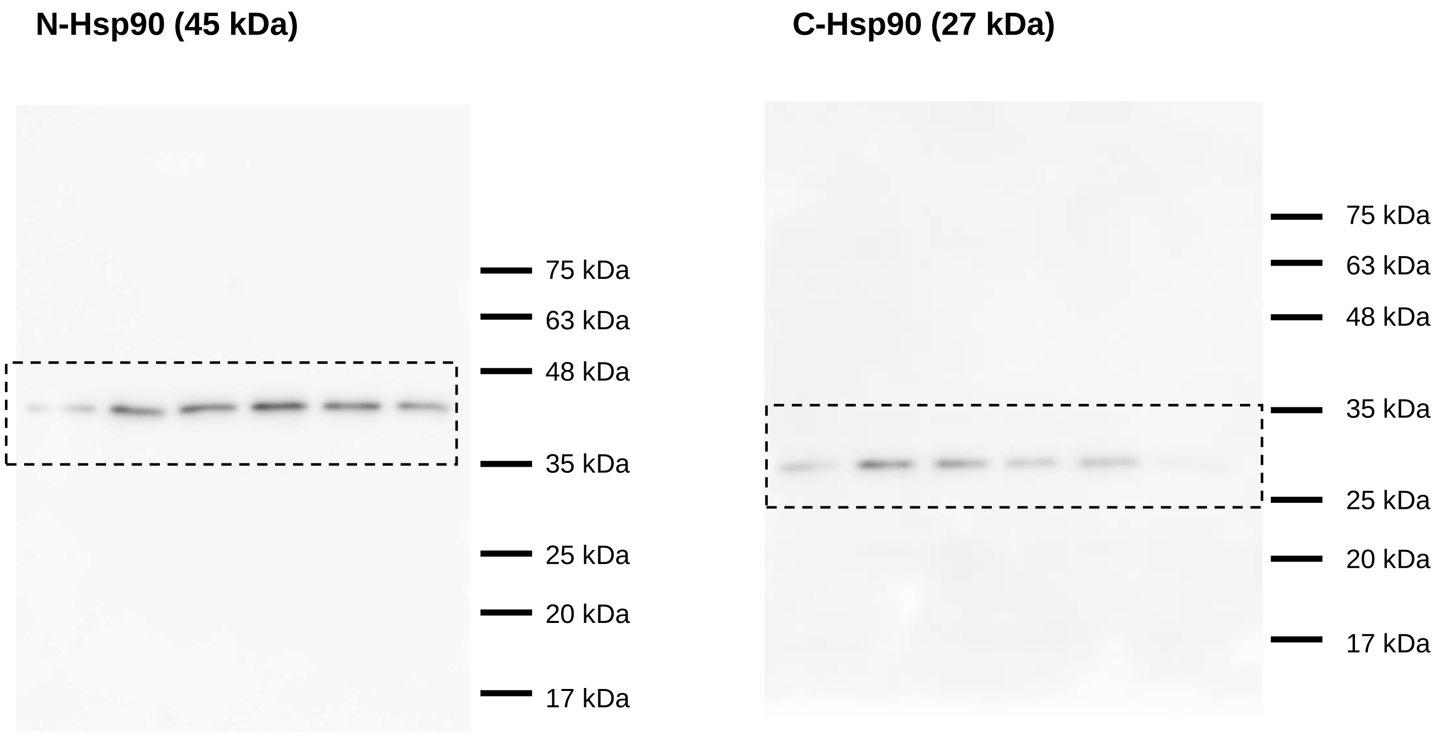
N-Hsp90 (45 kDa)
75 kDa
63 kDa
48 kDa
35 kDa
25 kDa
20 kDa
17 kDa
C-Hsp90 (27 kDa)
75 kDa
63 kDa
48 kDa
35 kDa
25 kDa
20 kDa
17 kDa
